# A Neutrality Test for Detecting Selection on DNA Methylation Using Single Methylation Polymorphism Frequency Spectrum

**DOI:** 10.1093/gbe/evu271

**Published:** 2014-12-23

**Authors:** Jun Wang, Chuanzhu Fan

**Affiliations:** Department of Biological Sciences, Wayne State University

**Keywords:** epigenetics, epimutation, neutrality test, single methylation polymorphism, site frequency, Tajima’s *D*

## Abstract

Inheritable epigenetic mutations (epimutations) can contribute to transmittable phenotypic variation. Thus, epimutations can be subject to natural selection and impact the fitness and evolution of organisms. Based on the framework of the modified Tajima’s *D* test for DNA mutations, we developed a neutrality test with the statistic “*D*^m^” to detect selection forces on DNA methylation mutations using single methylation polymorphisms. With computer simulation and empirical data analysis, we compared the *D*^m^ test with the original and modified Tajima’s *D* tests and demonstrated that the *D*^m^ test is suitable for detecting selection on epimutations and outperforms original/modified Tajima’s *D* tests. Due to the higher resetting rate of epimutations, the interpretation of *D*^m^ on epimutations and Tajima’s *D* test on DNA mutations could be different in inferring natural selection. Analyses using simulated and empirical genome-wide polymorphism data suggested that genes under genetic and epigenetic selections behaved differently. We applied the *D*^m^ test to recently originated *Arabidopsis* and human genes, and showed that newly evolved genes contain higher level of rare epialleles, suggesting that epimutation may play a role in origination and evolution of genes and genomes. Overall, we demonstrate the utility of the *D*^m^ test to detect whether the loci are under selection regarding DNA methylation. Our analytical metrics and methodology could contribute to our understanding of evolutionary processes of genes and genomes in the field of epigenetics. The Perl script for the “*D*^m^” test is available at http://fanlab.wayne.edu/ (last accessed December 18, 2014).

## Introduction

Epigenetic marks, such as DNA methylation, have been shown to affect gene expression (Zhang et al. 2006; Zilberman et al. 2007; Laurent et al. 2010; Zemach et al. 2010; Li et al. 2012). Similar to spontaneous nucleotide mutations in DNA sequences, spontaneous errors can also occur in epigenetic modifications (epimutation) (Becker et al. 2011; Schmitz et al. 2011; Schmitz, Schultz, et al. 2013). These epimutations can be transgenerationally inherited and accumulate over an evolutionary timescale, resulting in heritable phenotypic variations (Bender and Fink 1995; Cubas et al. 1999; Manning et al. 2006; Heijmans et al. 2008; Martin et al. 2009; Becker et al. 2011; Hitchins et al. 2011; Schmitz et al. 2011; Calarco et al. 2012; Hirsch et al. 2012; Jiang et al. 2013; Schmitz, Schultz, et al. 2013; Silveira et al. 2013; Cortijo et al. 2014; Dias and Ressler 2014). Thus, epimutations can contribute to the fitness of organisms, and the evolution of epimutations could be driven by natural selection. For example, organisms can adapt to variable environments/ecological niches by way of epigenetic variations (Rapp and Wendel 2005; Geoghegan and Spencer 2012, 2013; Hirsch et al. 2012). Therefore, it would be imperative to detect the loci whose epigenetic modifications are under natural selection, in order to enhance our understanding of the evolutionary dynamics of organisms from an epigenetic aspect.

DNA methylation mutation, which is defined as a methyl group being added or removed from a cytosine base in DNA, is one type of inheritable epimutations. As more and more whole-genome base resolution methylome data of populations become available, the intraspecific single methylation polymorphisms (SMPs) can be used to investigate the evolutionary processes of DNA methylation (Becker et al. 2011; Schmitz et al. 2011; Eichten et al. 2013; Heyn et al. 2013; Schmitz, He, et al. 2013; Schmitz, Schultz, et al. 2013). Studies have shown that SMPs have the following four properties: 1) Although C-differentially methylated regions (C-DMRs) and genetic variants may be linked, association between SMPs/CG-DMRs and genetic variants was rarely observed (Schmitz, Schultz, et al. 2013); 2) the epimutations rate ($\mu_{m}$) is comparatively high. The methylation mutation rate with the lower bound of 4.46 × 10^−^^4^/(cytosine of CG)/generation is orders of magnitude greater than the DNA mutation rate, which is around 1 × 10^−^^8^ to 10^−^^9^/base/generation (Ossowski et al. 2010; Schmitz et al. 2011). Thus, the epimutation parameter $\theta_{m}$ based on SMPs ($\theta_{m}=4N\mu_{m}$_,_ N is the effective population size, $\mu_{m}$ is the epimutation rate per generation) is theoretically greater than mutation parameter (θ) based on single nucleotide polymorphisms (SNPs) (θ=4Nμ_,_ μ is the mutation rate per generation). 3) The loci bearing SMPs are finite. The percentage of methylated cytosines (mCs) among all the cytosines in *Arabidopsis* is about 75% (Seymour et al. 2014); and 4) the epimutation rate varies among different cytosine sites. Only a small proportion of mCs (approximately 10% over whole genome and approximately 40% in gene body of *Arabidopsis*) show epimutations. Among them, a few sites experience rapid epimutations (Becker et al. 2011; Schmitz et al. 2011). Previously, limited studies investigated natural selection on DNA methylation. Using chimpanzee as the outgroup to search for the CG-cytosine sites bearing the methylation state with single human population outliers, a recent report identified positively selected CG-cytosine sites in human populations (Heyn et al. 2013).

Tajima’s *D* test (*D*) is a classic neutrality test to detect natural selection in nucleotide sequences (Tajima 1989). Basically, *D* compares the difference between two estimators of the mutation parameter θ(θ=4Nμ), and normalizes the difference with its standard deviation. θ can be estimated by the average number of pairwise nucleotide differences (π) and by the number of segregation sites (S) among a sample of DNA sequences. Under neutral scenarios, the two estimators, $\theta_{\pi }$ and $\theta_{\text{s}}$, should have similar values and their difference should be around zero. However, natural selection or demographic effects (e.g., population expansion or shrinkage) can influence the allele frequency and give rise to a biased allele frequency spectrum. $\theta_{\pi }$ takes into account of the allele frequency spectrum, but $\theta_{\text{s}}$ does not. Therefore, natural selection or demographic effects influence the two estimators differently, which can lead to nonzero difference. Later, based on *D* (Tajima 1989), Misawa and Tajima developed a modified Tajima’s *D* test (*D*^mod^) by deriving the equations of the $\theta_{\pi }$ and $\theta_{\text{s}}$ estimators (Misawa and Tajima 1997) following three assumptions: 1) Mutations occur in finite sites, 2) the neutral mutation rate varies among sites, and 3) θ can be large (Tajima 1989; Misawa and Tajima 1997).

These assumptions mostly fit the aforementioned properties of DNA methylation mutations. However, *D*^mod^ is used for the mutations in DNA sequences, where each site has four possible nucleotide states (A, T, C, or G), whereas each cytosine has only two methylation states (methylated or unmethylated). Taking into consideration the SMP characteristics mentioned above and the SMP frequency spectrum, we developed a new test “*D*^m^” for methylation mutations based on the framework of *D* and *D*^mod^. Basically, we derived the equations of DNA methylation mutation parameter $\theta_{m}$ estimated by the average methylation state difference per site $\left(\pi_{m}\right)$ and by proportion of methylation segregation sites $\left(s_{m}\right)$. Then, we compared the difference of the two estimators and normalized the difference by its standard deviation. The difference could show whether a locus has an excess of low-frequency or intermediate-frequency SMPs, suggesting the signature of selection and/or demographic changes in DNA methylation. We applied *D*^m^ to SMPs simulated with an epigenetic inheritance model and under population epigenetic selection models. We compared *D*^m^ with *D* and *D*^mod^. The simulation results showed that *D*^m^ was capable of detecting selection on SMPs, although it could also be sensitive to demographic effects. We then applied *D*^m^ to empirical SMP data from *Arabidopsis* and human, and further analyzed whether there is any association between natural selection on SMPs and SNPs. Finally, we used *D*^m^ to detect natural selection on DNA methylation of newly evolved genes in *Arabidopsis* and human genomes. Overall, the results based on both simulated and empirical SMP data suggest the utility of *D*^m^ as a neutrality test for DNA methylation mutations.

## Materials and Methods

### Construction of the Neutrality Test

We assume, in a finite-site and Cavender–Farris–Neyman (CFN) model (Neyman 1971; Farris 1973; Cavender 1978) with equal methylation state frequencies and equal epimutation (gain or loss of methylation) rates, “n” DNA sequences are randomly sampled from the population. Thus, following the approach of Tajima’s (1996) work, we obtain the probability ($p_{i})$ of a particular cytosine site exclusively methylated or exclusively unmethylated in the sample as below:

(1) $$p_{i}=\frac{\Gamma \left(2\theta_{m}\right)\Gamma \left(\theta_{m}+n\right)}{\Gamma \left(\theta_{m}\right)\Gamma \left(2\theta_{m}+n\right)}.$$

We assume that the epimutation rate parameter, *θ_m_* ($4N\mu_{m})$ per cytosine site per generation, follows a gamma distribution (Kimura 1968; Ewens 1972; Schmitz et al. 2011; Berke et al. 2012):

(2) $$g\left(\theta_{m}\right)=\frac{\beta^{\alpha }}{\Gamma (\alpha )}e^{-\beta \theta_{m}}\theta_{m}{}^{\alpha -1}.$$

We denote *π_m_* as the average number of pairwise methylation state differences per cytosine site, and *s*_m_ as the proportion of segregating cytosine sites (the number of segregating sites per cytosine site). Following Tajima (Tajima 1996; Misawa and Tajima 1997), we obtained:

(3) $$E\left(s_{m}\right)\approx a_{1}*E\left(\theta_{m}\right)*\frac{1}{1+c_{1}\frac{\left(\alpha +1\right)}{\alpha }E\left(\theta_{m}\right)},$$

(4) $$E\left(\pi_{m}\right)\approx \frac{E\left(\theta_{m}\right)}{1+\frac{2\left(\alpha +1\right)E\left(\theta_{m}\right)}{\alpha }},$$

$$\begin{array}{c} a_{1}=\overset{n-1}{\underset{i=1}{\sum }}\frac{1}{i},\\ a_{2}=\overset{n-1}{\underset{i=1}{\sum }}\frac{1}{i^{2}},\\ a_{3}=\frac{{\left(a_{1}\right)}^{2}-a_{2}}{2},\\ c_{1}=2a_{1}-\frac{3a_{3}}{a_{1}}. \end{array}$$

Notably, the equation of $c_{1}$ and equation (4) above are different from those of $c_{1}$ and equation (22) in Tajima (1996).

After transformation and approximation:
(5)
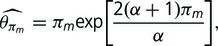

(6)
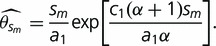



In Tajima’s *D* test (Tajima 1989),

$$\begin{array}{c} D=\frac{\pi -\frac{S}{a_{1}}}{\sqrt{e_{1}S+e_{2}S(S-1)}},\\ b_{1}=\frac{n+1}{3\left(n-1\right)},\\ b_{2}=\frac{2(n^{2}+n+3)}{9n(n-1)},\\ c_{3}=b_{1}-\frac{1}{a_{1}},\\ c_{4}=b_{2}-\frac{n+2}{a_{1}n}+\frac{a_{2}}{a_{1}^{2}},\\ e_{1}=\frac{c_{3}}{a_{1}},\\ e_{2}=\frac{c_{4}}{a_{1}^{2}+a_{2}}. \end{array}$$

π is the average number of nucleotide differences over the whole sequence, and S is the number of segregating sites over the whole sequence.

Substituting 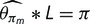 and 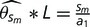 (*L* is the length of cytosines in the sequence) into above Tajima’s *D* test, we developed the neutrality test statistic for methylation mutations as
(7)
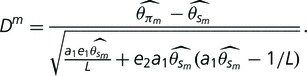



Under neutral hypothesis, $E\left(D^{m}\right)\approx 0$ and $V\left(D^{m}\right)\approx 1$, which were shown by the simulation results in the later section. The detailed derivation steps of these mathematical equations are described in supplementary material S1, Supplementary Material online.

### Computer Simulation

#### The Inheritance Model of DNA Methylation

Both theoretical and empirical studies showed that DNA methylation could be inherited in a way similar to genetic mutation (Richards 2008; Jablonka and Raz 2009; Slatkin 2009; Becker et al. 2011; Schmitz et al. 2011; Cortijo et al. 2014). At a specific cytosine site, we denote “1” as methylated and “0” as unmethylated. We denote the loss rate as “γ” and the gain rate as “δ.” Hence, the inheritance of epigenetic mark can be modeled using a two-state Markov chain. The transition rate matrix, Q, describing the instantaneous rate of change between 0 and 1, is

 01Q={qij}=01[−δδγ−γ].

The diagonals are specified by the requirement that each row of *Q* sums to zero. *Q* determines the transition probability matrix, *P*, over any time *t* > 0: $P\left(t\right)=\left\{p_{ij}\left(t\right)\right\}$ where $p_{ij}\left(t\right)$ is the probability that when *t* = 0, the site is the *i* state, and after *t* > 0, the site is the *j* state.

$$\frac{dP(t)}{dt}=P\left(t\right)Q.$$

Thus, $P\left(t\right)=e^{Qt}$, with the boundary condition P(0)=I, the identity matrix (when *t* = 0, no transition occurs). Therefore, the model of epigenetic inheritance is analogous to the one of DNA substitution in the finite sites. Here, we considered the simplest model when γ=δ=μ per generation and the initial distribution $\pi^{\left(0\right)}$ $=\left(\pi_{0}^{\left(0\right)},\pi_{1}^{\left(0\right)}\right)=\left(0.5,0.5\right)$, thus

$$\begin{array}{c} Q=\left\{q_{ij}\right\}=\left[\begin{array}{cc} -\mu & \mu \\ \mu & -\mu \end{array}\right]\text{ and }\\ P_{ij}\left(t\right)=\{\begin{array}{c} \frac{1}{2}+\frac{1}{2}{\text{e}}^{-2\mu t}ifi=j\\ \frac{1}{2}-\frac{1}{2}{\text{e}}^{-2\mu t}ifi\neq j \end{array}. \end{array}$$

Slatkin also proposed a model of epigenetic inheritance (Slatkin 2009). In contrast to the above one-parameter model assuming equal methylation gain and loss rate, Slatkin’s model uses two parameters to describe the rates of gain and loss, respectively. Simulations with both models generated similar results, supporting the utility of *D*^m^ (see supplementary material S2 for details of Slatkin’s model, supplementary figs. S1 and S2 and table S1, Supplementary Material online). However, the advantage of one-parameter model is the simplicity, which uses one parameter to describe epimutation rate. When the knowledge about the methylation gain rate and loss rate remains largely unknown, it is more practical and easier to implement one-parameter model. Therefore, we only present the results based on the above one-parameter model below.

#### Neutral Scenarios

We used “ms” package (Hudson 2002) to generate 10,000 random genealogy trees of 60 samples with coalescent algorithm assuming no recombination and no demographic effects. The branch length of the tree is the evolutionary time, *T*, between the ancestor and its child nodes, where *T* was measured in the unit of 4*N* generation (T=t/4N). Then, we modified the “seq-gen” package (Rambaut and Grassly 1997) to generate 1,000-bp sequences of methylation states evolving along these genealogy trees, according to the above inheritance model and epimutation (gain or loss of methylation) rates of different sites following a gamma distribution. We choose different parameter values in the modified “seq-gen” to allow the mean of $\theta_{m}$ and α to take on different values (see supplementary material S2, Supplementary Material online, for more details).

### Test Powers for Selection and Demographic Effect

Under different demographic scenarios and the population epigenetic selection models, we simulated SMPs with the mean of $\theta_{m}=0.1$ per site per generation, which is gamma distributed among the sites with α=0.5. All tests were one-sided. The test power was computed as the proportion of test statistic values falling into the lower or upper 5% tail of the null distribution (a neutral model without recombination, selection, and demographic effects). The parameters and commands for running “ms” and “ms_sel” package are listed in supplementary material S1, Supplementary Material online.

#### Demographic Effects

For population size changes, we used “ms” package to generate random genealogy trees assuming instantaneously 100 × population expansion or 1/100 population shrinkage occurring at different time points (measured from the present backwards in units of 4*N* generations) with no recombination. For population subdivision, we used “ms” to generate random genealogy trees assuming island model with symmetric migration rate between two subpopulations with 4*Nm* = 0.1, because the *D*^m^ test is only sensitive to strong subdivision, for example, 4*Nm* < 0.5 (Zeng et al. 2006). And we took different sampling schemes from the two subpopulations to test the sensitivity of the neutrality tests. Based on the generated genealogy trees of different demographic models, we used the modified “seq-gen” to generate the sequences of methylation states according to the above inheritance model.

#### Selection Models

We adopted the population epigenetic models of selection developed by Geoghegan and Spencer (2012) (“Model 1” and “Model 2”). According to Geoghegan and Spencer’s Model 1, which assumes a single autosomal locus A with two epialleles, A_1_ and A_2_ that could be epigenetically modified by two different environments (*j* = 1,2). They assigned the corresponding epiallele frequencies as $p_{1}$ and $p_{2}$, respectively, where ∑p_*k*_ = 1. The frequencies of epialleles A_1_ and A_2_ after selection_,_ $p_{1}^{s}$ and $p_{2}^{s}$_,_ can be calculated using Geoghegan and Spencer’s equations (1) and (2). Subsequently, the frequencies in the next generation can be computed using recursion equations as Geoghegan and Spencer’s equations (3) and (4). The mean fitness of population, $\bar{w},$ is the sum of $p_{\text{1}}^{\text{s}}$ and $p_{2}^{\text{s}}$ (Geoghegan and Spencer 2012) (see supplementary material S2, Supplementary Material online, for more details).

According to Geoghegan and Spencer’s Model 2, which assumes a single autosomal locus with two alleles and epialleles inherited from parents residing in two different environments (*j* = 1,2). A_j_ and a_j_ assign for epialleles “A” and “a”, respectively. The epiallele frequencies of A_1_, A_2_, a_1_ and a_2_ are *p*_1_, *p*_2_, *p*_3_ and *p*_4_, respectively, where $\sum^{\text{​}}p_{k}=1$. Similar to “Model 1,” the frequencies of epialleles A_1_, A_2,_ a_1_ and a_2_ after selection_,_ $p_{\text{1}}^{\text{s}}$_,_ $p_{\text{2}}^{\text{s}}$, $p_{\text{3}}^{\text{s}}$ and $p_{\text{4}}^{\text{s}}$, can be calculated using Geoghegan and Spencer’s equations (7)–(10). Subsequently, the frequencies in the next generation can be computed using recursion equations as Geoghegan and Spencer’s equations (11)–(14). The mean fitness of population, $\bar{w}=p_{1}^{\text{s}}+p_{\text{2}}^{\text{s}}+p_{\text{3}}^{\text{s}}+p_{\text{4}}^{\text{s}}$ (Geoghegan and Spencer 2012) (see supplementary material S2, Supplementary Material online, for more details).

The equilibrium solutions of Geoghegan and Spencer’s metrics are algebraically complicated. Thus, Geoghegan and Spencer (2012) explored the parameter space numerically. Based on their results, we picked several parameter settings, which show directional selection and heterozygote disadvantage in two environments, and permitted a stable equilibrium (as shown in figs. 6 and 11 of Geoghegan and Spencer’s article). Based on these parameter settings, we generated the frequency trajectories of epialleles/alleles. Based on the frequency trajectories, we generated the genealogy trees of 60 samples of the two epialleles/alleles using ms_sel package with coalescent algorithm, kindly provided by R. Hudson (University of Chicago).

The “ms_sel” program package simulates selective sweeps, and it has been applied in various studies (Coop and Ralph 2012; Leffler et al. 2013; Pavlidis et al. 2013). We first generated frequency trajectories of the selected alleles. Based on these frequency trajectories, ms-sel coalescently determined the genealogy tree of selection favored site and its linked sites. The rationale behind is that the distribution of ancestral frequencies of selected alleles (namely, the frequency trajectories here) can reveal the genealogy describing the states (a coalescence or mutation event) in the coalescence processes and the interval time between different states (Kaplan et al. 1988). The effect of recombination could also be included into the simulation of genealogy tree (Hudson and Kaplan 1988).

We assigned that the epigenetic sequence (that is defined as the sequence of DNA methylation states) length (*L*) is 1,000 bp whereas the number of breakpoints is 999 and recombination happens between adjacent base pairs. The population recombination rate (4N*r) is 8 × 10^−^^4^ that is consistent with that of *Arabidopsis* (Kim et al. 2007). Thus, recombination probability parameter is 4N*r*(*L* − 1) = 0.7992; and the 500th site evolves under selection. Then, using the modified “seq-gen”, we simulated the sequences of methylation states evolving along the above genealogy trees under “Model 1” and “Model 2.” Similarly, we assigned that the DNA sequence length is 4,000 bp (4*1,000 bp). The recombination probability parameter is 4N*r*(*L* − 1) = 3.1992, and the 2,000th site evolves under selection. Then, using the original “seq-gen,” we simulated the DNA sequences evolving along the genealogy trees under “Model 2” (see supplementary material S2, Supplementary Material online, for more details).

### Estimation α Value of Gamma Distribution

The α is the shape parameter of a gamma distribution by which we describe the distribution of mutation rate among cytosines in the *D*^m^ test. In a parsimony analysis, the distribution of the (minimum) numbers of changes per site will approximately follow a Poisson distribution if the change rate is constant, but will approximately follow a negative binomial distribution if the change rate is gamma distributed (Tamura and Nei 1993; Yang and Kumar 1996). To infer the (minimum) number of changes per site, it is necessary to have the phylogenetic tree for all the sequences first. This can be inferred using the neighbor-joining method (Tamura and Nei 1993). Further, the changes in the tree topology may have little effect when estimating the relative frequencies of nucleotide substitution (Tamura and Nei 1993).

Based on the empirical SMP data, we constructed a phylogenetic tree of all the samples using the neighbor-joining algorithm for each locus of interest. Then according to the constructed neighbor-joining tree and parsimony method, we counted the number of sites (*N_k_*) at which *k* changes are inferred along the tree when *k* ≤ 5, and assumed the rest of sites with *k* = 6 (for simplicity). As mentioned above, if the change rate is gamma distributed with shape parameter α, the distribution of *k* will follow a negative binominal distribution. Thus, the sample mean (*m*) and variance (*s*^2^) of *k* can be equated to the mean and variance of the negative binomial distribution, respectively, and parameter α can be estimated by

$$\alpha =m^{2}/(s^{2}-m)$$

(Tamura and Nei 1993). When the *s*^2^ is 0 (namely, no variance), we assumed α is 1,000,000. And when $s^{2}<m$, we assumed $\alpha =m^{2}/(s^{2}*\exp\left(-\frac{m}{s^{2}}\right))$ (substituting $1-\frac{m}{s^{2}}=\text{exp}(-\frac{m}{s^{2}})$ in the above equation). Thus, we estimated α for each locus of interest. We further analyzed the effect of overestimation or underestimation of α on the test.

### Collection of SMP and SNP Data of *Arabidopsis*

We downloaded the processed Methyl-C sequencing data of mixed stage inflorescence samples of 50 *Arabidopsis* accessions from National Center for Biotechnology Information (NCBI) Gene Expression Omnibus (GEO) under accession number GSE43857: http://www.ncbi.nlm.nih.gov/geo/query/acc.cgi?acc=GSE43857 (Schmitz, Schultz, et al. 2013). We then collected the SMPs on the cytosines mapped in all the 50 *Arabidopsis* accessions. We further extracted CG-SMPs from C-SMPs, because gene body usually has a higher abundance of mCG (Becker et al. 2011; Li et al. 2012; Schmitz, Schultz, et al. 2013). We downloaded the processed and quality filtered SNP data of the same *Arabidopsis* accessions as the SMP data from http://signal.salk.edu/atg1001/download.php. The gene annotation “.gff” file of *Arabidopsis* was obtained from Phytozome v8.0 (http://www.phytozome.net/) with *Arabidopsis thaliana* 167 (TAIR release 10 acquired from TAIR at http://www.arabidopsis.org/download/index.jsp).

### Collection of Human SMP and SNP Data

We downloaded the processed HumanMethylation450 BeadChip data of the lymphoblastoid cells from NCBI GEO under accession number GSE36369: http://www.ncbi.nlm.nih.gov/geo/query/acc.cgi?acc=GSE36369. This data set includes 96 individuals from African-American population, 96 individuals from Asian-American population, and 96 individuals from Caucasian Americans (Heyn et al. 2013). Notably, we removed 11 Caucasian and 8 African individuals who were identified as outliers by Heyn et al. (2013). We only considered the probes in the gene body region. To simulate the diploid format of the human genome, we approximated the methylation states of each mapped CG-cytosine site according to its β value, which is equal to methylation signal/(methylation signal + unmethylation signal + 100) (Heyn et al. 2013). Sites with β < 0.33 were annotated as unmethylated and unmethylated; those with β > 0.66 were represented as methylated and methylated; and those in the between were annotated as unmethylated and methylated (Heyn et al. 2013). We then collected the SMPs on the CG-cytosines mapped in each of the three human populations. The corresponding relationship between genes and chip probes was based on the Illumina description file, GPL13534_HumanMethylation450_15017482_v1.1.csv, downloaded from http://www.ncbi.nlm.nih.gov/geo/query/acc.cgi?acc=GPL13534. The reference gene annotation was based on “refGene.txt” downloaded from UCSC genome browser http://hgdownload.cse.ucsc.edu/goldenPath/hg19/database/. We also downloaded the processed SNP data of the same three human populations (African-American, Asian-American, and Caucasian-American population) from NCBI GEO under accession number GSE24245 (Niu et al. 2010). These data included the SNP mapped in 96 individuals of each of the three human populations with Illumina HumanExon510-Sv1 DNA BeadChip.

### The Randomly Selected Gene Sets

A species with complicated demography often has a skewed frequency spectrum. This causes these population genetics test statistics to be far more negative/positive than are expected under a standard neutral model. It, thus, leads to false positive in search for footprints of adaptive events if a standard neutral model is used as the null hypothesis. To overcome this, we performed alternative population genetics analysis using empirical distribution from a large amount of unlinked genes in the genome, which has been tested previously (Schmid et al. 2005; Toomajian et al. 2006; Ramos-Onsins et al. 2008; Wang et al. 2013). We randomly picked 2,425 genes across the entire *Arabidopsis* genome, each of which has at least one SMP among the 50 accessions, as *D*, *D*^mod^, and *D*^m^ could not be computed if no SMP is present. Two randomly selected genes (RGs) are at least 25 kb apart from each other as linkage disequilibrium in *A. thaliana* decays on average within 25–50 kb (Nordborg et al. 2005; Ramos-Onsins et al. 2008; Schmitz, Schultz, et al. 2013). We randomly chose 6,178–6,271 genes across the human genome except sex chromosomes due to that sex chromosome has different effective population size from the autosomes, each of which has at least one SMP among all the individuals of each of three subpopulations. Two RGs are at least 100 kb apart from each other to avoid the possible linkage disequilibrium in human genome (Bosch et al. 2009). Based on these RG selected criteria, approximately 2,000 and approximately 6,000 are the maximum numbers of RGs that we can generate in *Arabidopsis* and human genomes, respectively.

## Results

### Validation of the D^m^ Test through Simulated SMP Data under Neutral Mode

We developed the equations of two estimators, 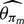 and 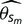, to estimate $\theta_{m}$. We then constructed *D*^m^ based on the difference between the two estimators (see eqs. 5 and 6). To validate *D*^m^ and further compare it with *D* and *D*^mod^, we simulated SMP data assuming that equal frequency of methylation and unmethylation, and the rates of spontaneous methylation gain and loss are equal as $\theta_{m}$ (epimutation rate), and the epimutation rates among sites follow a gamma distribution with the shape parameter alpha α (Schmitz et al. 2011; Berke et al. 2012). We applied and calculated *D*^m^, *D,* and *D*^mod^ to the simulated SMP data by considering different $\theta_{m}$ and α values in neutral mode without selection, recombination, and demographic effects (see Materials and Methods for the details).

**Fig. 1.—:**
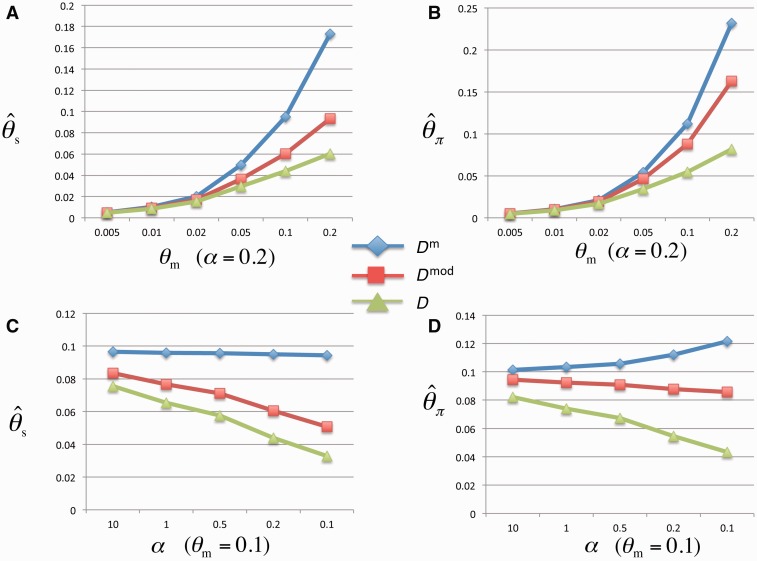
Comparison of the $\theta_{m}$ estimators from the *D*^m^, *D*^mod^, and *D* tests based on the simulated SMP data. (*A*) The *x* axis is the simulated $\theta_{m}$ value with α fixed at 0.2; and the *y* axis is the estimated $\theta_{m}$ value from the $\hat{\theta_{s_{m}}}$ estimators of the three tests. (*B*) The *x* axis is the same as (*A*); and the *y* axis is the estimated $\theta_{m}$ value from the $\hat{\theta_{\pi_{m}}}$ estimators of the three tests. (*C*) The *x* axis is the simulated α value with $\theta_{m}$ fixed at 0.1, and the *y* axis is the same as (*A*). (*D*) The *x* axis is the same as (*C*), and the *y* axis is the same as (*B*).

**Fig. 2.—:**
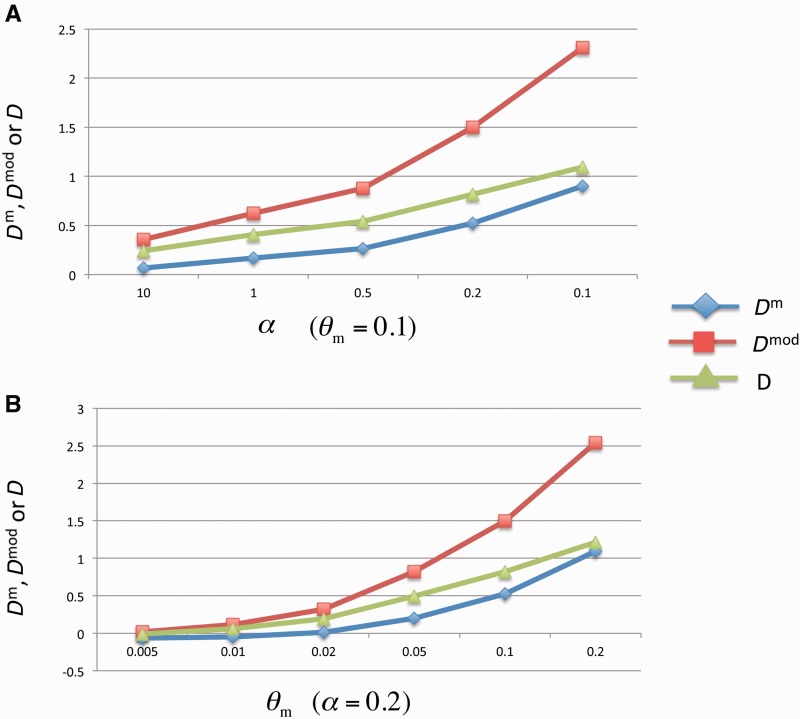
Comparison of the test statistics of the *D*^m^, *D*^mod^, and *D* tests based on the simulated SMP data. (*A*) The *x* axis is the simulated α value with $\theta_{m}$ fixed at 0.1, and the *y* axis is the *D*^m^, *D*^mod^ or *D* value. (*B*) The *x* axis is the simulated $\theta_{m}$ value with α fixed at 0.2, and the *y* axis is the same as (*A*).

The means of $\hat{\theta_{\pi_{m}}}$ and $\hat{\theta_{s_{m}}}$ from *D*^m^ are closer to the assumed $\theta_{m}$ values than those of the estimators from *D* and *D*^mod^ for all the considered parameter values. The biases of the estimates from *D* and *D*^mod^ are more evident when the assumed $\theta_{m}$ value increases and α value decreases (supplementary table S2, Supplementary Material online, and fig. 1). Moreover, compared with *D*^mod^ and *D*, the mean of *D*^m^ is closer to zero, suggesting that the *D*^m^ test is the best metrics as a neutrality test for epimutations (supplementary table S3, Supplementary Material online, and fig. 2). Cautiously, we observed that $\hat{\theta_{s_{m}}}$ also underestimates $\theta_{m}$ and $\hat{\theta_{\pi_{m}}}$ overestimates $\theta_{m}$ when the assumed $\theta_{m}$ value increases and α value decreases (supplementary table S2, Supplementary Material online, and fig. 1). Two factors could cause these deviations. First, the epimutation rate is gamma distributed. When we assumed larger $\theta_{m}$ and smaller α value to simulate the SMPs, a few sites, which experienced multiple epimutation turnarounds (gain and loss of methylation), ended up as fewer methylation changes. This leads to that the number of segregation sites decrease and $\hat{\theta_{s_{m}}}$ underestimate $\theta_{m}$. Second, there are only two methylation states. When epimutation rate (the assumed $\theta_{m}$) is high, the proportion of intermediate-frequency SMPs will increase, leading to that $\hat{\theta_{\pi_{m}}}$ will overestimate $\theta_{m}$.

We further simulated the situations with unequal initial frequencies of methylation and unmethylation for two cases: 1) The case with equal methylation gain and loss rate and 2) the case where methylation gain and loss rate depending on the epimutation rate and equilibrium frequencies of methylation and unmethylation frequencies. For these situations, *D*^m^ still performed better than *D*^mod^ and *D* (see supplementary material S2 for more details, supplementary figs. S3 and S4, Supplementary Material online).

### Test Sensitivity for Demographic Effects

#### Population Growth

**Fig. 3.—:**
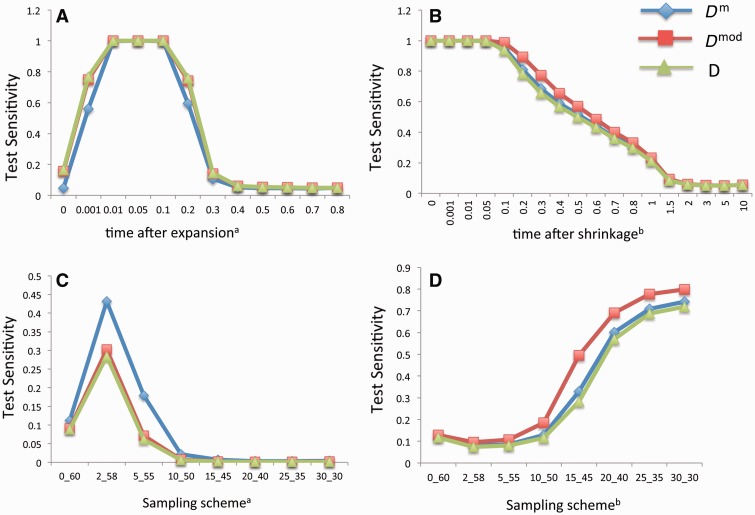
Comparison of the sensitivity of the *D*^m^, *D*^mod^, and *D* tests to different demographic effects based on the simulated SMP data. (*A*) For population expansion, *x* axis is the time period after the instantaneous population growth, measured in the unit of *4N* generations. (*B*) For population shrinkage, *x* is the time period after the instantaneous population shrinkage, measured in the unit of *4N* generations. (*C*, *D*) For population subdivision model, *x* axis is the sampling scheme of 60 samples. The *y* axis is the test power of the three tests. ^a^The test power was computed as the proportion of test statistic values falling into the lower 5% tail of the null distribution. ^b^The test power was computed as the proportion of test statistic values falling into the upper 5% tail of the null distribution.

Population growth can cause an excess of low-frequency SMPs, leading to the test statistics of *D*^m^, *D*^mod^, and *D* shifting to the negative values of the null distribution. As shown in figure 3*A*, all three tests are sensitive to the population growth (the population size instantaneously increases 100 times) soon after it occurs, and remain sensitive until 0.4*4*N* generations, and their sensitivities are similar.

#### Population Shrinkage

When population size decreases, the proportions of intermediate-frequency SMPs will increase and the proportions of low-frequency SMPs will decrease, leading to the test statistics of *D*^m^, *D*^mod^, and *D* shifting to the positive values of the null distribution. With the population size instantaneously decreasing 100 times, the sensitivities of all three tests are at the similar levels, which stay around 1 before 0.1*4*N* generation, decrease afterwards, and disappear after 1.5*4*N* generations (fig. 3*B*).

#### Population Subdivision

The effect of population subdivision with low migration level (e.g., 4N*m < 0.5, *m* as the migration rate) is similar to the effect of high mutation rate. When the sampling scheme is biased, there are a large proportion of low-frequency SMPs. As the sampling scheme becomes more even, the proportion of intermediate-frequency SMPs will increase. Therefore, under population subdivision scenarios, the test statistics of *D*^m^, *D*^mod^, and *D* shift from the negative values of the null distribution to the positive values as the sampling scheme becomes more even. All three tests are sensitive to population subdivision at similar levels in either negative or positive direction (fig. 3*C* and *D*).

### Test Power for Detecting Selection

The theoretical studies by Geoghegan and Spencer revealed that in population epigenetic selection models, the fixation of epialleles is not at stable equilibrium as epigenetic variations could be constantly regenerated (Geoghegan and Spencer 2012, 2013). Specifically, under directional selection in epialleles, when the epigenetic resetting rate (which is the frequency of resetting to the epiallele type induced by the residing environment, see supplementary material S2, Supplementary Material online) is low (<0.5), an excess of low-frequency polymorphisms can be observed. This is analogous to a genetic directional selection scenario when the fixation is approaching but not complete yet. Although, when epigenetic resetting rate is high (≥0.5) and the frequencies of epialleles residing in different environments are equal, a higher proportion of common polymorphisms can be maintained even under epigenetic heterozygote disadvantage (Geoghegan and Spencer 2012, 2013). This is analogous to the genetic balancing selection scenario. To validate the powers of the three tests in detecting selection of epimutations, we simulated SMPs under the two population epigenetic selection models proposed by Geoghegan and Spencer: “Model 1” assumes a monoallele and biepiallele locus; and “Model 2” assumes a biallele and biepiallele locus (Geoghegan and Spencer 2012).

**Fig. 4.—:**
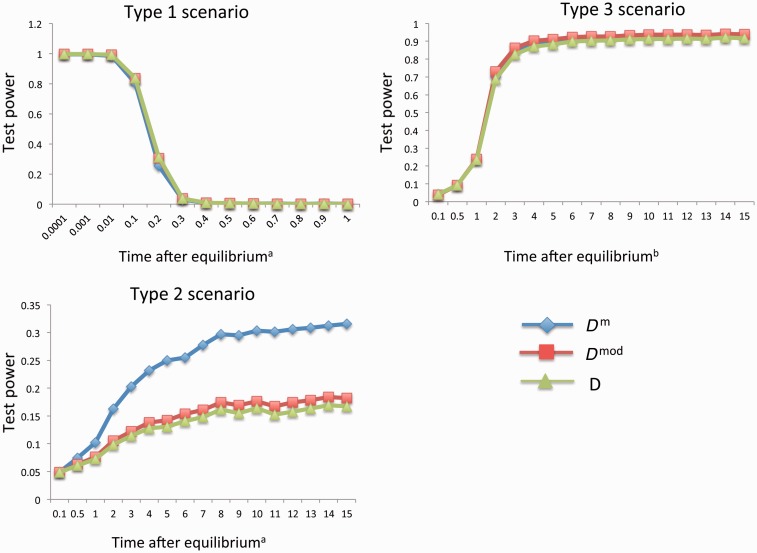
Comparison of the *D*^m^, *D*^mod^, and *D* tests in detecting selection based on the SMP data simulated under the population epigenetic selection “Model 1.” The *x* axis is the time period after epialleles arrive at the stable equilibrium, measured in the unit of 4*N* generations. The *y* axis is the test power. ^a^The test power was computed as the proportion of test statistic values falling into the lower 5% tail of the null distribution. ^b^The test power was computed as the proportion of test statistic values falling into the upper 5% tail of the null distribution. See the main text for the description of the three types of scenarios.

Based on “Model 1,” we simulated the three types (Type 1, Type 2, and Type 3) of epiallelic frequency trajectories in the two situations where the epiallele frequencies are approaching and have been maintained at equilibrium for certain evolutionary time, respectively (see Materials and Methods, and supplementary fig. S5, Supplementary Material online). “Type 1” trajectories consider that the frequency of one epiallele decreases from 1 to 0.05–0.1 and that of the other increases from 0 to 0.9–0.95. “Type 2” trajectories consider that the frequency of one epiallele decreases from 1 to 0.9–0.95 and that of the other increases from 0 to 0.05–0.1. “Type 3” trajectories consider that the frequency of one epiallele decreases from 1 to 0.4–0.5 and that of the other increases from 0 to 0.5–0.6. At the moment when the equilibrium just arrives, all three tests (*D*^m^, *D*^mod^, and *D*) can detect the selection in Type 1 and Type 3 scenarios in the negative direction with similar powers (table 1). When the equilibrium has been maintained for certain evolutionary time, for the Type 1 scenario, the test powers are in the negative direction and decrease as time lasts. All three tests performed at the same power level (fig. 4).

**Table 1:** Test Powers in the Three Scenarios under the Population Epigenetic Selection “Model 1”

*r*	*t*	Initial		Equilibrium		Scenario Type	Test Power		
		*p*_1_	*p*_2_	*p*_1_	*p*_2_		*D*^m^	*D*^mod^	*D*
0.5	0.1	0	1	0.1093	0.8907	2	0.0467	0.0467	0.0474
0.5	0.1	1	0	0.1093	0.8907	1	0.9948	0.9971	0.9974
0.5	0.5	0	1	0.413	0.587	3	0.124	0.1317	0.1322
0.5	0.5	1	0	0.413	0.587	3	0.3213	0.3421	0.3464
0.5	0.9	0	1	0.5103	0.4897	3	0.2049	0.2166	0.2187
0.5	0.9	1	0	0.5103	0.4897	3	0.1921	0.2045	0.2055
0.1	0.5	0	1	0.0552	0.9448	2	0.0457	0.0457	0.0455
0.1	0.5	1	0	0.0552	0.9448	1	0.9999	1	1
0.9	0.5	0	1	0.9378	0.0622	1	1	1	1
0.9	0.5	1	0	0.9378	0.0622	2	0.043	0.0432	0.0435

Note.—*r*, the frequency of environment where epialleles reside; *t*, the probability of epigenetic resetting to the epigenetic state in the residing environment.

For the Type 2 scenario, when the equilibrium frequency of one epiallele is around 0.05, the selection becomes detectable in the negative direction and *D*^m^ performed much better than *D*^mod^ and *D* (fig. 4). As shown in equations (5), (6), $c_{1}$ and figure 1, *D* and *D*^mod^ tests underestimate $\hat{\theta_{\pi_{m}}}$ and $\hat{\theta_{s_{m}}}$, which is severer in *D* test, and have a stronger effect on $\hat{\theta_{s_{m}}}$ than $\hat{\theta_{\pi_{m}}}$, which is severer in *D*^mod^ (supplementary table S4, Supplementary Material online). When a large excess of rare epialleles exist in the Type 2 scenario (one epiallele was maintained in the frequency of 0.05–0.1), $\hat{\theta_{\pi_{m}}}$ is smaller than $\hat{\theta_{s_{m}}}$, leading the test statistic *D*^m^, *D*^mod^, and *D* toward negative direction. However, compared with *D*^m^ test, the severe underestimation of $\hat{\theta_{s_{m}}}$ and $\hat{\theta_{\pi_{m}}}$ in *D* leads the test statistic closer to the neutral null distribution, whereas much stronger underestimation on $\hat{\theta_{s_{m}}}$ than $\hat{\theta_{\pi_{m}}}$ in *D*^mod^ leads the test statistic to be less negative (supplementary table S4, Supplementary Material online). Therefore, the final distributions of *D*^mod^ and *D* in Type 2 scenario are closer to the neutral null distributions than that of *D*^m^, leading to *D*^mod^ and *D* tests having lower power than *D*^m^ test. In contrast, in Type 1 scenario, there is a much larger excess of very rare epialleles (data not shown), $\hat{\theta_{\pi_{m}}}$ is much smaller than $\hat{\theta_{s_{m}}}$, leading the test statistic *D*^m^, *D*^mod^, and *D* toward negative direction in far enough distance (supplementary table S4, Supplementary Material online). Thus, the underestimation of $\hat{\theta_{s_{m}}}$ and $\hat{\theta_{\pi_{m}}}$ by *D* and *D*^mod^ have relatively weak impact on the difference between *D*^mod^/*D* distributions in Type 1 scenario and neutral scenario. So *D*^mod^ and *D* tests have similar test power level in Type 1 scenario compared with *D*^m^ test.

For the Type 3 scenario, the test powers to detect selection are in the positive direction and at the same level (fig. 4). Because a much larger excess of intermediate epialleles (data not shown) exist in Type 3 scenario, $\hat{\theta_{\pi_{m}}}$ is much larger then $\hat{\theta_{s_{m}}}$, leading the test statistic *D*^m^, *D*^mod^, and *D* toward positive direction in far enough distance (supplementary table S4, Supplementary Material online). Therefore, severe underestimation on $\hat{\theta_{s_{m}}}$ and $\hat{\theta_{\pi_{m}}}$ in *D* and stronger underestimation on $\hat{\theta_{s_{m}}}$ than $\hat{\theta_{\pi_{m}}}$ in *D*^mod^ have relatively weak impact on the difference between *D*^mod^/*D* distributions in Type 3 scenario and neutral scenario.

**Fig. 5.—:**
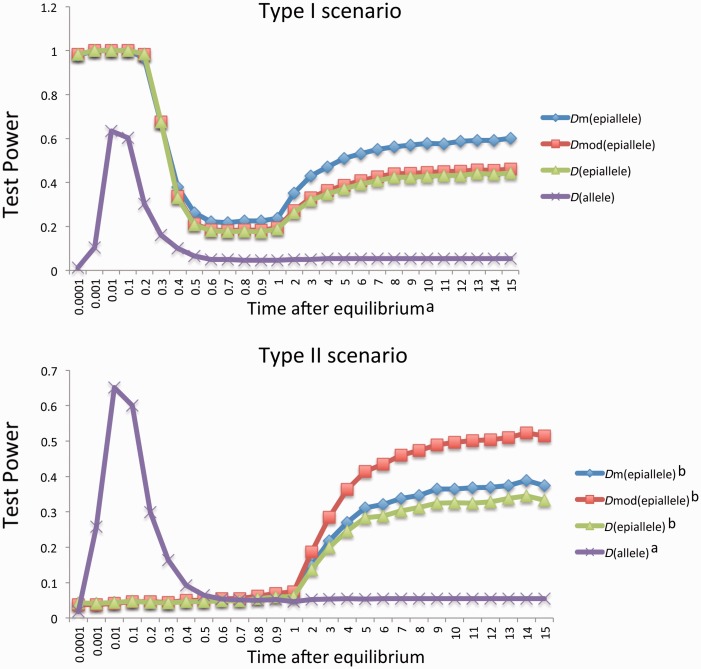
Comparison of the *D*^m^, *D*^mod^, and *D* tests in detecting selection based on the SMP data and *D* based on SNP data, which are simulated under the population epigenetic selection “Model 2.” The *x* axis is the time period after epialleles arrive at the stable equilibrium, measured in the unit of 4*N* generations. The *y* axis is the test power. ^a^The test power was computed as the proportion of test statistic values falling into the lower 5% tail of the null distribution. ^b^The test power was computed as the proportion of test statistic values falling into the upper 5% tail of the null distribution. See the main text for the description of the two types of scenarios.

Based on “Model 2,” we generated two types (Type I and Type II) of allele and epiallele frequency trajectories in the two situations where the epiallele frequencies are approaching and have been maintained at equilibrium for certain evolutionary time, respectively (see Materials and Methods, and supplementary fig. S2, Supplementary Material online). Both Type I and Type II consider the existence of two alleles and one epiallele initially but have different frequency trajectories for the new deriving epiallele. For Type I, the frequency of the new deriving epiallele rises from 0 to 0.9972 when the frequency of one of alleles increases from 0.8 to 1. For Type II, the frequency of the new deriving epiallele rises from 0 to 0.2436 when the frequency of one of alleles increases from 0.8 to 1. When the equilibrium is approaching, *D* can detect selection for alleles in both Type I and Type II scenarios in the negative direction, whereas *D*^m^, *D*^mod^, and *D* can detect selection on epialleles in the negative direction only for Type I scenario with similar levels of power (table 2). After equilibrium arrives, for Type I scenario, *D* is powerful for detecting selection in the negative direction on alleles only during a short time window (e.g., 0.001∼0.4*4*N* generation after fixation), whereas *D*^m^, *D*^mod^, and *D* are powerful for detecting selection on epialleles in the negative direction for most of time. Furthermore, *D*^m^ outperformed *D*^mod^ and *D* after 0.4*4*N* generations (fig. 5). The polymorphism spectrum after 4*N* generation in the Type I scenario will be similar to that in the Type 2 scenario of “Model 1”, namely, a large excess of rare epialleles are observed (data not shown). Therefore, the better performance of *D*^m^ than *D*^mod^ and *D* tests in that stage can be explained by the aforementioned reason for Type 2 scenario of “Model 1” (supplementary table S4, Supplementary Material online).

**Table 2:** Test Powers in the Two Scenarios under the Population Epigenetic Selection “Model 2”

*r*	*t*	Initial		Equilibrium		Scenario Type	Test Power		
		*P_A_*	*P_a_*	*P_A_*	*P_a_*		*D*^m^	*D*^mod^	*D*
		*P*_1_	*P*_2_	*P*_1_	*P*_2_				
0.1	0.2	0.2	0.8	0	1	I			0.511
		1	0	0.0028	0.9972		0.9804	0.9835	0.9835
0.33	0.2	0.2	0.8	0	1	II			0.518
		1	0	0.7564	0.2436		0.0691	0.0711	0.0708

Note.—*r*, the frequency of environment where epialleles reside; *t*, the probability of epigenetic resetting to the epigenetic state in the residing environment.

For Type II scenario, similar to the case in Type I, *D* is powerful for detecting selection on genetic variants in the negative direction during a short evolutionary period, whereas *D*^m^, *D*^mod^, and *D* become powerful for detecting selection on epialleles in positive direction later (e.g., 2*4*N* generation after equilibrium arrives). The performance rank of the three tests is *D*^mod^ > *D*^m^ > *D* (fig. 5). Because Type II scenario generates an excess of intermediate frequency epialleles, $\hat{\theta_{\pi }}$ is larger then $\hat{\theta_{s}}$, leading the test statistic *D*^m^, *D*^mod^, and *D* toward positive direction. Compared with *D*^m^ test, the intense underestimation of $\hat{\theta_{s_{m}}}$ and $\hat{\theta_{\pi_{m}}}$ by *D* leads the test statistic closer to the neutral null distribution, whereas much stronger underestimation of $\hat{\theta_{s_{m}}}$ than $\hat{\theta_{\pi_{m}}}$ by *D*^mod^ leads the test statistic farther toward positive direction (supplementary table S4, Supplementary Material online). Therefore, the final distribution of *D* in Type II scenario is closer to the neutral null distribution than that of *D*^m^, and *D* test has lower test power than *D*^m^ test. And the final distribution of *D*^mod^ stays farther positive from the neutral null distribution than that of *D*^m^, thus *D*^mod^ displays higher test power than *D*^m^.

### The Effect of α to the D^m^ Test

The empirical survey of SMPs among the *Arabidopsis* lines, which shared a common ancestor 30 generations ago, suggested that methylation mutation rate varies among the cytosine sites. The majority of cytosines did not have SMPs and fewer SMPs had a higher frequency of occurrence (Schmitz et al. 2011; Berke et al. 2012). Therefore, similar to *D*^mod^, we assumed that the epimutation rate among the cytosine sites of a locus is gamma distributed with the shape parameter α. We applied “the method of moments” to estimate α for each of the loci when analyzing empirical data (see Materials and Methods) (Tamura and Nei 1993). This method, however, may not accurately estimate α. For example, depending on the phylogenetic tree of the sampled sequences, α could be overestimated when α is small (Yang and Kumar 1996; Misawa and Tajima 1997). Therefore, we evaluated the effect of overestimation and underestimation of α on *D*^m^ with simulations.

First, we simulated SMPs with α = 0.1, 0.2, 1, and 10 under neutral scenario, we then analyzed the distribution of *D*^m^, *D*^mod^, and *D* computed with α = 0.5 based on the SMPs (supplementary table S5, Supplementary Material online). When α is overestimated, the distribution of *D*^m^ moves toward the positive direction with a smaller variance. When α is underestimated, the distribution of *D*^m^ moves toward the negative direction with a larger variance. This is due to an overestimation of α leading to underestimation of $\hat{\theta_{\pi_{m}}}$ and $\hat{\theta_{s_{m}}}$, whereas underestimation of α can overestimate $\hat{\theta_{\pi_{m}}}$ and $\hat{\theta_{s_{m}}}$. The bias effects are stronger on $\hat{\theta_{s_{m}}}$ than $\hat{\theta_{\pi_{m}}}$ (referred to eqs. 5, 6, and $c_{1}$ parameter). Regardless, we still found that the means of *D*^m^ are more close to zero compared with *D*^mod^ and *D* in most cases.

Second, we simulated SMPs with α=0.5 under selection model, we then computed the power of *D*^m^ with α= 0.05 or 10 based on the SMPs. For the aforementioned three types of selection scenarios in “Model 1,” overestimation of α did not impact the power much, but underestimation of α weakened the power (supplementary table S6, Supplementary Material online). Therefore, if α cannot be precisely estimated, it is better to be overestimated than to be underestimated.

### Application D^m^ to Empirical SMP Data

**Fig. 6.—:**
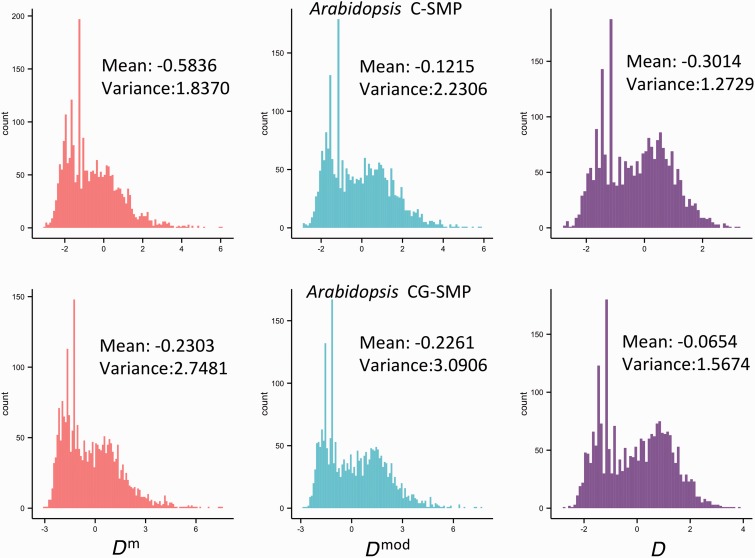
Comparison of the *D*^m^, *D*^mod^, and *D* tests based on the empirical SMP data of *Arabidopsis*.

**Fig. 7.—:**
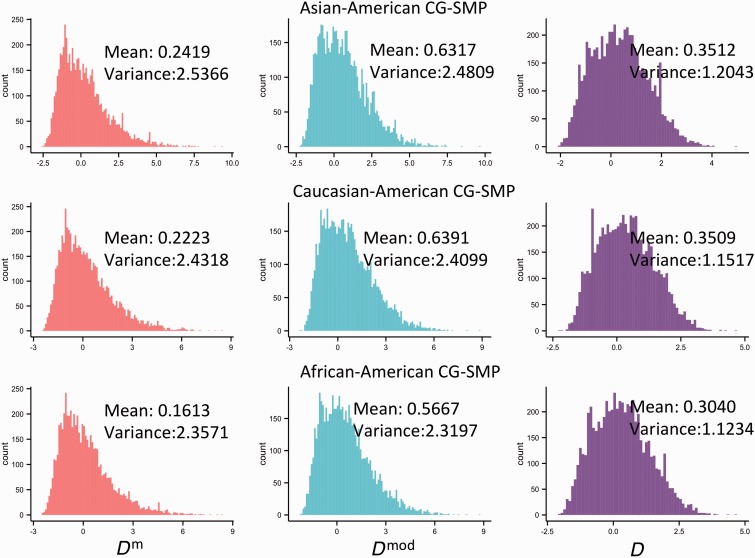
Comparison of the *D*^m^, *D*^mod^, and *D* tests based on the empirical SMP data of human.

We applied the *D*^m^ test to C-SMPs and CG-SMPs of approximately 2,400 RGs in the *Arabidopsis* genome, and to CG-SMPs of approximately 6,200 RGs in the human genome (see Materials and Methods). The distributions of *D*^m^, *D*^mod^, and *D* for all RGs were shown in figures 6 and 7. The *D*^m^ values are more negative than those of *D*^mod^ and *D* (Wilcoxon rank sum test, *P* < 10^−^^12^). As mentioned above, *D* considers mutations occurring in an infinite site model and *D*^mod^ considers four nucleotide types in a finite site model. Thus, both *D* and *D*^mod^ tests would underestimate θ for epimutations ($\hat{\theta_{\pi_{m}}}$ and $\hat{\theta_{s_{m}}}$) that occur between two methylation states and in finite sites (fig. 1). Moreover, the underestimation will have a stronger effect on $\hat{\theta_{s_{m}}}$ than $\hat{\theta_{\pi_{m}}}$ when $\theta_{m}$ and the sample size are larger, and the α value is smaller (fig.1, and referred to eqs. 5, 6, and $c_{1}$ parameter), pushing the distribution of *D*^mod^ and *D* toward the positive direction. In addition, we found that the *D*^m^ of African-American population is significantly different from Asian-American and Caucasian-American populations (Wilcox rank sum test, *P* value < 0.05), but no significant difference between Asian-American population and Caucasian-American population was observed, suggesting that the SMP frequency spectrum may differ among different human populations.

Additionally, the distributions of *D*^m^, *D*^mod^, and *D* do not resemble a standard neutral model in *Arabidopsis*. This observation may not be due to a majority of genes being under positive or balancing selection on DNA methylation, but rather could be explained by pervasive purifying selection, the selfing nature, and the complex demographic history of *Arabidopsis* (Innan and Stephan 2000)*.* Both positive and purifying selection can generate excesses of rare epialleles, leading to *D*^m^, *D*^mod^, and *D* moving toward negative direction, thus we cannot distinguish the two types of selection from *D*^m^, *D*^mod^, and *D* values. And purifying selection may work more pervasively in the genome than positive selection (Slotte et al. 2011). Demographic effects have been found contributing to the nonstandard neutral distribution of *Arabidopsis* SNPs (Schmid et al. 2005) and may have a stronger impact on SMP frequency spectrum due to the higher epimutation rate. Therefore, to account for the possible pervasive purifying selection and the demographic effects, it is necessary to use an empirical distribution of test statistics from a large randomly selected data set in the genome, which avoids linkage disequilibrium, as the null distribution of the neutrality test (Nordborg et al. 2005; Schmid et al. 2005; Wright and Gaut 2005; Toomajian et al. 2006).

However, randomly selected genes include some nonneutral loci where the loci were influenced under positive, purifying or balancing selection. We used simulation to estimate the effect of test power if empirical distributions include a large proportion of nonneutral RGs. We simulated 1,000 genes under each of three types of selection scenarios in “Model 1” and 7,000 genes under the neutral mode (total 10,000 genes). With the statistic distribution of the 10,000 genes as the null distribution, we repeated the analyses of the test power for selection. We found that the powers of the three tests are indeed decreased when compared with our previous results (supplementary table S7 and fig. S7, Supplementary Material online). Therefore, we need to be cautious that the use of empirical distributions may also increase false negative rate and detect fewer events with selection footprints (purifying or positive selection).

### Comparison of Neutrality Tests Based on SNPs and SMPs

We analyzed whether there is an association between the selection on SMPs detected by *D*^m^ and that on SNPs detected by *D*. We used the maximum same set of 21,885 *Arabidopsis* genes to compute *D* values with SNPs from the same 50 *Arabidopsis* accessions as SMP data. We used the maximum set of 9,172 genes with both mapped SMPs and SNPs in African-American population, 14,771 genes in Caucasian-American population, and 14,407 genes in Asian Americans. We used the empirical distribution of the above RGs as the null distribution to compute the critical values (with 5% Type I error for one tail).

In *Arabidopsis*, using *D*^m^ test, we identified 1,312 and 1,028 genes falling into the 5% lower and upper tails of the null distribution of SMPs, respectively. Using *D* test, we identified 1,079 and 1,201 genes falling into the 5% lower and upper tails of the null distribution of SNPs, respectively. Among them, 220 genes showed selection signatures in both genetic (SNP) and epigenetic (SMP) levels (table 3). If selection on DNA mutations and epimutations occurs completely independently, we would expect to observe 244 genes with selection signature in both genetic and epigenetic levels ([1,312 + 1,028]/21,885 in SMPs* [1,079 + 1,201]/21,885 in SNPs *21,885 genes = 244 genes). Thus, the observed number of genes with both epigenetic and genetic selection signatures (220 genes) is not different from the random expectation (244 genes, binomial test, *P* = 0.13). Further, some genes fell into 5% tail of null distribution for SMPs and SNPs in the same direction, whereas other genes fell in the opposite direction (table 3).

**Table 3:** The Number of Genes Falling in 5% Lower and 5% Upper Tails of the Null Distribution of SNPs and SMPs and the Number of Genes Overlapped among the Four Categories in *Arabidopsis* and Three Human Populations

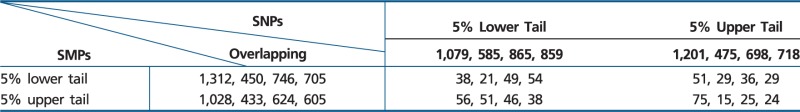

Note.—The number order as *Arabidopsis,* African American, Caucasian American, Asian American.

Additionally, when we applied *D*^mod^ and *D* to this empirical SMP data, compared with *D*^m^ test, *D*^mod^ and *D* identified 72 (6%) and 38 (3%) fewer genes falling into the 5% lower tail of the null distribution, respectively. This empirical analysis is consistent with our previous simulation results showing that *D*^m^ outperforms *D*^mod^ and *D* in detecting the loci with excesses of rare epialleles.

In human, using *D*^m^ test, we identified 450 and 433 genes falling into the 5% lower and upper tails of the null distribution of SMPs in African-American population, 746 and 624 in Caucasian-American population, and 705 and 605 in Asian-American population, respectively (table 3). Based on *D* test, we detected 585 and 475 genes falling into the 5% lower and upper tails of the null distribution of SNPs in African-Americans, 865 and 689 in Caucasian-Americans, and 859 and 718 in Asian-Americans, respectively (table 3). The numbers of overlapped genes bearing the outlier (falling into the 5% lower or upper tails of the null distribution) *D*^m^ and *D* values are similar to random expectations and binomial test reported insignificant *P* value (*P* value: African American: 0.1633, Caucasian American: 0.315, Asian American: 0.8667). This result is similar to the above one in *Arabidopsis*.

### Selection Driving the Methylation Variation of Newly Evolved Genes

Changes of epigenetic modifications have been hypothesized and shown as a fast and powerful mechanism to preserve newly originated genes through silencing or differentiating their expression from their parental sequences (Rodin and Riggs 2003; Adams et al. 2004; Silveira et al. 2013). To verify this hypothesis and detect whether such processes were driven by natural selection, we applied *D*^m^ to the SMPs of two sets of lineage-specific new genes.

The first data set includes the *Arabidopsis* lineage-specific new duplicate genes (NDGs) and their parental genes (PGs) (Wang et al. 2013). Among them, 40 NDGs and 45 PGs have C-SMP data, and 37 NDGs and 43 PGs have CG-SMP data. We applied *D*^m^ to their C-SMPs and CG-SMPs. After computing *D*^m^ for NDGs and PGs, we compared the distribution of their *D*^m^ with those of the RGs. We found that for C-SMPs and CG-SMPs, the *D*^m^ values of NDGs were significantly smaller than those of RGs (Wilcoxon rank sum test, one tailed *P* < 0.005; table 4), whereas no significant difference of *D*^m^ was observed between PGs and RGs (table 4). We also found a larger proportion of NDGs falling into the lower 5% tail of null SMP distribution than those of PGs and RGs (table 4), implying prevalent natural selection on NDGs. Alternatively, recently evolved genes might encounter a relaxed purifying selection during their early stage to accumulate an excess of deleterious epimutations, so a higher proportion of NDGs may be observed with excesses of rare-SMPs. Among the 12 NDGs with significant *D*^m^ values (in the lower or upper 5% tail of null distribution), 11 genes have annotated functional roles (supplementary table S8, Supplementary Material online). Overall, the results show that newly evolved genes contain higher number of rare methylation variants, suggesting that selection may act on the epimutations of new genes during their early formation processes.

**Table 4:** Comparison of the *D*^m^ Values of NDGs and PGs with Those of RGs

SMP Types	Mean *D*^m^	Variance of *D*^m^	Wilcox test *p*^a^	No. (%) of Genes^b^
RG_C_SMP	−0.5836	1.8370		
NDG_C_SMP	−1.3076	2.0000	1.577e-05	10 (25.00)
PG_C_SMP	−0.6740	1.6025	0.3732	4 (8.89)
RG_CG_SMP	−0.2303	2.7481		
NDG_CG_SMP	−0.8595	2.9818	0.002072	6 (16.22)
PG_CG_SMP	0.0851	3.0276	0.8956	3 (6.98)

^a^The one-tailed *P* values were generated by comparing the *D*^m^ values of NDGs and PGs with those of RGs, respectively, using Wilcoxon rank sum test.

^b^The number of genes with an excess of rare-SMPs is based on empirical *P* values ≤ 0.05.

Additionally, when we applied *D*^mod^ and *D* to the SMP data, both *D*^mod^ and D tests identified three (43%) fewer NDGs and two (100%) fewer PGs falling into the 5% lower tail of the null distribution than *D*^m^, respectively; and *D* test recognized three (43%) fewer NDGs and two (100%) fewer PGs than *D*^m^. This result further demonstrated the better performance of *D*^m^ in detecting loci with excesses of rare-SMPs than *D*^mod^ and D.

The second data set includes eight hominoid-specific de novo genes (*MYEOV, MGC45800, C20orf166, TDRG1, FLJ26850, C19orf48, LOC284837*, and *C1orf229*)*,* which were recently identified by Xie et al. (2012). The eight genes were chosen because they still exist in the current Ensembl human gene annotation and are overlapped with human CG-SMP data generated by Heyn et al. (2013). Using the CG-SMP data from three human populations, we identified two de novo genes, *C19orf48* and *FLJ26850*, with excesses of low-frequency SMPs in Caucasian-American population but not the other two populations, which might suggest population-specific selection footprints. The *D*^m^ value of *C19orf48* equals −1.7829 (*P* = 0.0343), and the *D*^m^ value of *FLJ26850* equals −1.9863 (*P* = 0.0142). The *P* value was the proportion of RGs with *D*^m^ less than *D*^m^ of the gene of interest. As the *P* value is computed with the empirical distribution of RGs as the null, which may contain some selective loci, we may underestimate the significance level. Notably, both the genes were expressed in testis (Xie et al. 2012). We collected the annotation of the new genes in *Arabidopsis* and human with significant *D*^m^ values in the supplementary table S8, Supplementary Material online.

Finally, among 246 genes involved in population-specific CpG sites (pop-CpG) identified by Heyn et al. (2013), 11 genes (*MRPS18A, CLCN7, INPP5A, MCPH1, CDK11A, HSF1, SLC22A16, TSSC1, SERTAD3, NPHP4,* and *RNL*S) overlapped with the genes showing significant negative *D*^m^ (the genes falling in the lower 5% of null distribution among all the genes) in at least one population. Among 27 genes bearing pop-CpG sites with outlier characteristics of local selection identified by Heyn et al. (2013) (using Chimpanzee as the outgroup), four genes (*CDK11A*, *SLC22A16, NPHP4,* and *RNLS*) were overlapped with significant negative *D*^m^ genes (the genes falling in the lower 5% of null distribution among all the genes) in at least one population. The small number of overlapping genes can be explained by the different methodologies and objectives between two analyses. Specifically, we used the SMP frequency spectrum in the entire gene body within one population in the test, but Heyn et al. identified any single cytosine site bearing differential population methylation pattern by compared the methylation level among three populations and used chimpanzee as the outgroup for the local selection analysis. Further, they considered intergenic, promoter and gene body regions (Heyn et al. 2013), but we only considered gene body regions.

## Discussion

Epimutations have been shown to be the transmittable information from one generation to the next that affects the traits and fitness of offspring, so natural selection could act on population experiencing changes of epigenetic marks. Thus, similar to infer the selection on DNA variations at the population level, intraspecific epimutation polymorphism spectrum could provide clues to detect the signature of natural selection on epialleles. Based on Tajima’s approaches (Tajima 1996; Misawa and Tajima 1997), we derived two estimators ($\hat{\theta_{\pi_{m}}}$ and $\hat{\theta_{s_{m}}}$) to compute the methylation mutation parameter *θ_m_*. Simulation results showed that both estimators could precisely estimate *θ_m_* when *θ_m_* is small. With the difference between $\hat{\theta_{\pi_{m}}}$ and $\hat{\theta_{s_{m}}}$, we developed the *D*^m^ test to detect natural selection on DNA methylation following the framework of Tajima’s *D* test (Tajima 1989). The basic rationale of the *D*^m^ test is that natural selection can influence the frequency spectrum of DNA methylation polymorphism, which could be detected by the difference of 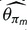 and 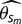. This rationale is supported by our simulation results.

However, theoretical studies and simulations also showed that directional selection in DNA methylation could cause an excess of intermediate-frequency epialleles (positive *D*^m^) or rare epialleles (negative *D*^m^) dependent on epimutation rate. Under a high epimutation rate, the frequency of an epiallele matching the dominant environment could be higher at equilibrium regardless of natural selection (Geoghegan and Spencer 2012). This contrasts with effects of selection on DNA variations where balancing selection generates an excess of intermediate-frequency alleles (positive D) and directional selection generates an excess of rare alleles (negative D). Therefore, the interpretation of *D*^m^ on SMPs could be different from that of *D* on SNPs. Namely, either extreme negative or positive *D*^m^ value could suggest directional selection on epialleles. Nevertheless, despite different interpretations of the implications of *D*^m^ on SMPs, simulations based on the population epigenetic selection models showed that the *D*^m^ test is capable of detecting selective signals of epimutations, and the power of *D*^m^ in detecting selection on methylation is comparable to the original or modified Tajima’s *D* test when they were directly applied to SMP data. All three tests can detect the footprints of selection that result in recently arising high-frequency methylation polymorphisms and long-lasting intermediate-frequency methylation polymorphisms. However, *D*^m^ performs substantially better than the other two tests in recognizing selection causing the long-lasting rare epialleles, which was further demonstrated by empirical SMP data analyses.

By applying the *D*^m^ test to empirical SMPs and the *D* test to empirical SNPs in *Arabidopsis*, we found that the number of genes with selection signatures in both SNPs and SMPs is close to the random expectation. Therefore, this result does support that genetic and epigenetic variations are rarely linked, which is consistent with the previous finding that SMPs are largely independent of SNPs (Schmitz, Schultz, et al. 2013). However, it is possible that tests of *D* on SNPs and *D*^m^ on SMPs are unable to ultimately detect selection footprints on both epigenetic and genetic levels at the same evolutionary time, which was also suggested by the simulations based on the population epigenetic selection model for a biallele and biepiallele locus (Model 2). Meanwhile, frequency spectrum tests based on SMPs and SNPs identified 100–200 genes with potential selection signatures in both genetic and epigenetic levels (table 3), suggesting that a small set of loci may have epigenetic variations that are closely associated with genetic variations. Overall, our analysis demonstrated that genetic and epigenetic variations could be subject to selection independently, though a small set of loci may behave as obligatory epigenetic variations that are completely associated with genetic variations (Richards 2006, 2008).

As showed in simulation, directional selection in DNA methylation can cause an excess of intermediate-frequency epialleles or rare epialleles dependent on epimutation rate. This contrasts with effects of selection on DNA variations where balancing selection generates an excess of intermediate-frequency alleles and directional selection generates an excess of rare alleles. Therefore, the genes with excesses of intermediate-frequency SNPs and SMPs may undergo different types of selective pressures on genetic (e.g., balancing selection) and epigenetic (e.g., directional selection) levels. In contrast, the genes with an excess of intermediate-frequency SMPs but an excess of rare SNPs may experience the same type of selective pressure (e.g., directional selection) on genetic and epigenetic levels.

We envision that the methodology of *D*^m^ can be broadly applicable as more and more genome-wide SMP data from different species become available, enhancing our understanding of evolutionary processes in the light of epigenetics. We acknowledge that *D*^m^ test can be improved in several aspects. First, better approaches are necessary to estimate the α parameter of *D*^m^. Second, to model the pattern of epimutations, we considered a simple symmetric CFN two-state model (Neyman 1971; Farris 1973; Cavender 1978) in *D*^m^, assuming equal frequencies of methylated and un-mCs, equal rate of methylation loss and gain, and an epimutation rate that follows a gamma distribution among sites. A sophisticated nonsymmetric epimutation model based on the empirical frequency and epimutation rate is also needed to further improve the neutrality test for epimutations.

## Supplementary Material

Supplementary materials S1 and S2, figures S1–S7, and tables S1–S8 are available at *Genome Biology and Evolution* online (http://www.gbe.oxfordjournals.org/).

Supplementary Data
