# Supplementary material for: A Neutrality Test for Detecting Selection on DNA Methylation Using Single Methylation Polymorphism Frequency Spectrum
Source: Genome Biol Evol. 2014 Dec 23;7(1):154–71. doi: 10.1093/gbe/evu271 (PMC4316624; doi:10.1093/gbe/evu271)
Supplement: Supplementary Data [file supp_7_1_154__index.html]

A Neutrality Test for Detecting Selection on DNA Methylation Using Single Methylation Polymorphism Frequency Spectrum — A Neutrality Test for Detecting Selection on DNA Methylation Using Single Methylation Polymorphism Frequency Spectrum — Supplementary Data 

# A Neutrality Test for Detecting Selection on DNA Methylation Using Single Methylation Polymorphism Frequency Spectrum

## Supplementary Data

files

**Files in this Data Supplement:**

- Supplementary Data - docx file
- Supplementary Data - docx file
