# Supplementary material for: A Neutrality Test for Detecting Selection on DNA Methylation Using Single Methylation Polymorphism Frequency Spectrum
Source: Genome Biol Evol. 2014 Dec 23;7(1):154–71. doi: 10.1093/gbe/evu271 (PMC4316624; doi:10.1093/gbe/evu271)
Supplement: Supplementary Data [file supp_evu271_New_Microsoft_Office_Word_Document.docx]

**Supporting Information**

**Supplementary Materials S1.** The derivation steps of equations for the D^m^ test

**Supplementary Materials S2.** The simulation of epigenetic data.

**Figure S1.** Comparison of the $\theta_{m}$ estimators from the D^m^, D^mod^ and D tests based on the simulated SMP data with Slatkin’s Model to the expected values.

**Figure S2.** Comparison of the test statistics of the D^m^, D^mod^ and D tests based on the simulated SMP data with Slatkin’s Model.

**Figure S3.** Comparison of the $\theta_{m}$ estimators and test statistics from the D^m^, D^mod^ and D tests under the model with unequal initial methylation and unmethylation frequency but equal methylation gain and loss rate.

**Figure S4.** Comparison of the $\theta_{m}$ estimators and test statistics from the D^m^, D^mod^ and D tests under the model with unequal initial methylation and unmethylation frequency and the methylation gain and loss rate depending on equilibrium methylation and unmethylation frequency.

**Figure S5**. The frequency trajectory of the A_1_ epiallele in the three types of scenarios under the population epigenetic selection “Model 1” developed by Geoghegan and Spencer.

**Figure S6**. The frequency trajectories of the allele A & a, and epiallele 1 & 2 in the two types of scenarios under the population epigenetic selection “Model 2” developed by Geoghegan and Spencer.

**Figure S7.** The D^m^, D^mod^ and D test powers in detecting selection based on the SMP data simulated under the population epigenetic selection “Model 1”, with the mixture of neutral loci and selective loci as the null distribution.

**Table S1.** The methylation gain ($\delta$) and loss ($\gamma$) rate applied in the simulations of Slatkin’s model

**Table S2.** The means of $\hat{\theta_{s}}$ and $\hat{\theta_{\pi}}$ from the three tests based on 10,000 simulation data under the neutral scenario.

**Table S3.** The means and standard deviations of D^m^, D^mod^, and D under the neutral scenario.

**Table S4.** The $\theta_{m}$estimator and test statistics of the three tests in the selection scenarios

**Table S5.** The means and standard deviations of D^m^, D^mod^, and D computed with $\alpha$=0.5 based on the SMPs simulated with $\alpha$=0.1, 0.2, 0.5, 1,10

**Table S6.** The means and standard deviations of D^m^, D^mod^, and D computed with $\alpha$=0.05 and 10 based on the SMPs simulated with $\alpha$=0.5

**Table S7.** The test powers in the three types of scenarios under the population epigenetic selection “Model 1” with the mixture of neutral loci and selective loci as the null distribution

**Table S8.** The annotation of the new genes in *Arabidopsis* and human with significant D^m^ values
