# Supplementary material for: A Neutrality Test for Detecting Selection on DNA Methylation Using Single Methylation Polymorphism Frequency Spectrum
Source: Genome Biol Evol. 2014 Dec 23;7(1):154–71. doi: 10.1093/gbe/evu271 (PMC4316624; doi:10.1093/gbe/evu271)
Supplement: Supplementary Data [file supp_evu271_Supplementary_materials.docx]

**Supplementary Materials S1**

**The derivation steps of equations for the D^m^ test**

*Note, the crucial equations that are different from the D or D^mod^ test are highlighted in red.*

Denoting the epimutation rate as per cytosine per generation by $\mu_{m}$ and the relative frequency of methylation state “i” in a particular cytosine site in the population by $\chi_{i}$ (methylated or un-methylated), and assuming there are “K” (K=2 here) possible states for one site, the probability distribution of $\chi_{i}$ is given by

$$\phi\left( \chi_{i} \right)=\frac{\Gamma\left( \alpha+\beta\right)}{\Gamma\left( \alpha\right)\Gamma\left( \beta\right)}{(1-\chi_{i})}^{\gamma-1}{\chi_{i}}^{\beta-1},$$

$\alpha=4N_{e}\mu_{m}$ and $\beta=\frac{4N_{e}\mu_{m}}{K-1}$ according to the equation 3.5 of Kimura 1968 paper [[1](#_ENREF_1)].

Here, we donated *θ_m_* as the epimutation parameter, $4N_{e}\mu_{m}=\theta_{m}$ and K =2

for methylation mutation:

$$\phi\left( \chi_{i} \right)=\frac{\Gamma\left( 2\theta_{m} \right)}{\Gamma\left( \theta_{m} \right)\Gamma\left( \theta_{m} \right)}{(1-\chi_{i})}^{\theta_{m}-1}{\chi_{i}}^{\theta_{m}-1} (1)$$

Supposing that “n” DNA sequences are randomly sampled from the population and using the Ewens sampling theory [[2](#_ENREF_2)], the probability ($p_{i})$ that a particular cytosine site in the sample is exclusively occupied by the methylation state “i” is given by

$$p_{i}=\int_{0}^{1} \chi_{i}^{n}\phi\left( \chi_{i} \right)d\chi$$

Based on equation (1) of $\phi\left( \chi_{i} \right)$, we obtain:

$$p_{i}=\int_{0}^{1} \frac{\Gamma\left( 2\theta_{m} \right)}{\Gamma\left( \theta_{m} \right)\Gamma\left( \theta_{m} \right)}\left( 1-\chi_{i} \right)^{\theta_{m}-1}\chi_{i}^{\theta_{m}-1}*\chi_{i}^{n}d\chi$$

$$=\frac{\Gamma(2\theta_{m})}{\Gamma(\theta_{m})\Gamma(\theta_{m})}\int_{0}^{1} {(1-\chi_{i})}^{\theta_{m}-1}\chi_{i}^{\theta_{m}+n-1}d\chi$$

Since beta function: $B\left( \chi+y \right)=\int_{0}^{1} t^{x-1}{(1-t)}^{y-1}dt$, we obtain

$$p_{i}=\frac{\Gamma\left( 2\theta_{m} \right)}{\Gamma\left( \theta_{m} \right)\Gamma\left( \theta_{m} \right)}B(\theta_{m}+n,\theta_{m})$$

Applying the relationship between beta function and gamma function:

$$B\left( \chi+y \right)=\frac{\Gamma\left( \chi\right)\Gamma\left( y \right)}{\Gamma\left( \chi+y \right)}$$

we have:

$$p_{i}=\frac{\Gamma\left( 2\theta_{m} \right)}{\Gamma\left( \theta_{m} \right)\Gamma\left( \theta_{m} \right)}*\frac{\Gamma\left( \theta_{m}+n \right)\Gamma\left( \theta_{m} \right)}{\Gamma\left( 2\theta_{m}+n \right)}$$

$$p_{i}=\frac{\Gamma(2\theta_{m})\Gamma(\theta_{m}+n)}{\Gamma(\theta_{m})\Gamma(2\theta_{m}+n)} (2)$$

Assuming “$s_{m}$” is the proportion of segregating cytosine sites (note: $s_{m}$is different from the number of segregating cytosine sites, which needs to be divided by the total number of cytosines in a locus to get $s_{m}$)[[3](#_ENREF_3)], and the probability ($p_{i})$ that a particular cytosine site in the sample is exclusively methylated equals to it is exclusively un-methylated, we have:

$$E(s_{m})=1-\sum_{i=1}^{2} p_{i}=1-2p_{i}$$

which is a modification of the equation 6 of Tajima 1996 paper [[3](#_ENREF_3)].

Because of the gamma function definition:

$$\Gamma\left( Z \right)=1*2*3*\ldots(Z-2)*(Z-1)$$

then

$$\Gamma\left( 2\theta_{m} \right)=1*2*3*\ldots*\left( 2\theta_{m}-2 \right)*\left( 2\theta_{m}-1 \right)$$

$$\Gamma\left( 2\theta_{m}+n \right)=1*2*3*\ldots*\left( 2\theta_{m}-2 \right)*\left( 2\theta_{m}-1 \right)*2\theta_{m}*\left( 2\theta_{m}+1 \right)*\ldots*\left( 2\theta_{m}+n-2 \right)*\left( 2\theta_{m}+n-1 \right)$$

$$\Gamma\left( \theta_{m} \right)=1*2*3*\ldots*\left( \theta_{m}-2 \right)*\left( \theta_{m}-1 \right)$$

$$\Gamma\left( \theta_{m}+n \right)=1*2*3*\ldots*\left( \theta_{m}-2 \right)*\left( \theta_{m}-1 \right)*\theta_{m}*\left( \theta_{m}+1 \right)*\ldots*\left( \theta_{m}+n-2 \right)*(\theta_{m}+n-1)$$

Therefore equation (2) becomes:

$$p_{i}=\frac{\theta_{m}*\left( \theta_{m}+1 \right)*\ldots*\left( \theta_{m}+n-2 \right)*(\theta_{m}+n-1)}{2\theta_{m}*\left( 2\theta_{m}+1 \right)*\ldots*\left( 2\theta_{m}+n-2 \right)*(2\theta_{m}+n-1)}$$

Based on unsigned Stirling numbers of the first kind:

$$({\chi)}^{\left( n \right)}=\chi*\left( \chi+1 \right)*\ldots*\left( \chi+n-1 \right)=\sum_{k=0}^{n} s(n,k)x^{k}$$

 thus

$$p_{i}=\frac{\sum_{k=0}^{n} S(n,k){(\theta_{m})}^{k}}{\sum_{k=0}^{n} S(n,k){(2\theta_{m})}^{k}}$$

Applying approximation:

$$\sum_{k=0}^{n} S\left( n,k \right){(\theta_{m})}^{k}\approx S\left( n,1 \right)\theta_{m}+S\left( n,2 \right){(\theta_{m})}^{2}+S\left( n,3 \right){(\theta_{m})}^{3}+\ldots$$

$$\sum_{k=0}^{n} S\left( n,k \right){(2\theta_{m})}^{k}\approx S\left( n,1 \right)2\theta_{m}+S\left( n,2 \right){4(\theta_{m})}^{2}+S\left( n,3 \right)8{(\theta_{m})}^{3}+\ldots$$

then,

$$E\left( s_{m} \right)=1-2p_{i}$$

$$\approx\frac{S\left( n,1 \right)2\theta_{m}+S\left( n,2 \right){4(\theta_{m})}^{2}+S\left( n,3 \right)8{(\theta_{m})}^{3}-2*(S\left( n,1 \right)\theta_{m}+S\left( n,2 \right){(\theta_{m})}^{2}+S\left( n,3 \right){(\theta_{m})}^{3})}{S\left( n,1 \right)2\theta_{m}+S\left( n,2 \right){4(\theta_{m})}^{2}+S\left( n,3 \right)8{(\theta_{m})}^{3}}$$

$$=\frac{S\left( n,2 \right){2(\theta_{m})}^{2}+S\left( n,3 \right){6(\theta_{m})}^{3}}{S\left( n,1 \right)2(\theta_{m})+S\left( n,2 \right){4(\theta_{m})}^{2}+S\left( n,3 \right)8{(\theta_{m})}^{3}}$$

$$E\left( s_{m} \right)\approx\frac{\frac{S\left( n,2 \right)\theta_{m}}{S(n,1)}+\frac{S\left( n,3 \right)3{(\theta_{m})}^{2}}{S(n,1)}}{1+\frac{S\left( n,2 \right)2(\theta_{m})}{S(n,1)}+\frac{S\left( n,3 \right)4{(\theta_{m})}^{2}}{S(n,1)}} (3)$$

since

$$S\left( n,1 \right)=1*2*\ldots*(n-1)$$

$$S\left( n,2 \right)=1*2*\ldots*\left( n-2 \right)+1*2*\ldots*\left( n-3 \right)*\left( n-1 \right)+1*2*\ldots*\left( n-4 \right)*\left( n-2 \right)*\left( n-1 \right)+\ldots$$

$$S\left( n,3 \right)=1*2*\ldots*\left( n-3 \right)+1*2*\ldots*\left( n-4 \right)*\left( n-2 \right)+1*2*\ldots*\left( n-4 \right)*(n-1)$$

Therefore

$$\frac{S(n,2)}{S(n,1)}=\frac{1}{n-1}+\frac{1}{n-2}+\ldots+1$$

$$\frac{S(n,3)}{S(n,1)}=\frac{1}{\left( n-1 \right)*(n-2)}+\frac{1}{\left( n-1 \right)*(n-3)}+\ldots+1$$

Let

$$a_{1}=\sum_{i=1}^{n-1} \frac{1}{i}=\frac{S(n,2)}{S(n,1)}$$

$$a_{2}=\sum_{i=1}^{n-1} \frac{1}{i^{2}}$$

$$a_{3}=\frac{\left( a_{1} \right)^{2}-a_{2}}{2}=\frac{S(n,3)}{S(n,1)}$$

Thus, equation (3) becomes:

$$E(s_{m})=1-2p_{i}\approx\frac{\frac{S\left( n,2 \right)\theta_{m}}{S(n,1)}+\frac{S\left( n,3 \right)3{(\theta_{m})}^{2}}{S(n,1)}}{1+\frac{S\left( n,2 \right)2\theta_{m}}{S(n,1)}+\frac{S\left( n,3 \right)4{{(\theta}_{m})}^{2}}{S(n,1)}}$$

$$=\frac{a_{1}\theta_{m}+a_{3}3{(\theta_{m})}^{2}}{1+a_{1}2\theta_{m}+a_{3}4{(\theta_{m})}^{2}}$$

$$=\frac{a_{1}\theta_{m}}{1+a_{1}2\theta_{m}-\left( a_{3}3{(\theta_{m})}^{2} \right)*\left( \frac{1+a_{1}2\theta_{m}}{a_{1}\theta_{m}} \right)+a_{3}4{(\theta_{m})}^{2}}$$

$$=\frac{a_{1}\theta_{m}}{1+2a_{1}\theta_{m}-\frac{3a_{2}\theta_{m}}{a_{1}}-a_{3}2{(\theta_{m})}^{2}}$$

$$E(s_{m})\approx\frac{a_{1}\theta_{m}}{1+(2a_{1}-\frac{3a_{2}}{a_{1}})\theta_{m}}$$

Assuming

$$c_{1}=2a_{1}-\frac{3a_{3}}{a_{1}}$$

then

$$E\left( s_{m} \right)\approx\frac{a_{1}\theta_{m}}{1+c_{1}\theta_{m}} (4)$$

Because the methylation mutation rate may vary among different cytosine sites, we assume the methylation mutation parameter $\theta_{m}$ is gamma distributed as the bellowing gamma distribution. Note that the smaller $\alpha$ is, the more the mutation rate varies among sites.

$$g\left( \theta_{m} \right)=\frac{\beta^{\alpha}}{\Gamma(\alpha)}e^{-\beta(\theta_{m})}{{(\theta}_{m})}^{\alpha-1}$$

which is the equation 19 of Tajima 1996 paper [[3](#_ENREF_3)].

And according to equation (4),

$$E\left( s_{m} \right)=\int_{0}^{\infty} \left( 1-2p_{i} \right)g\left( \theta_{m} \right)d\theta_{m}$$

$$\approx\int_{0}^{\infty} \frac{a_{1}\theta_{m}}{1+c_{1}\theta_{m}}*\frac{\beta^{\alpha}}{\Gamma(\alpha)}e^{-\beta{(\theta}_{m})}{{(\theta}_{m})}^{\alpha-1}d\theta_{m}$$

$$=\frac{a_{1}\beta^{\alpha}}{\Gamma(\alpha)}\int_{0}^{\infty} \frac{1}{1+c_{1}\theta_{m}}e^{-\beta(\theta_{m})}{{(\theta}_{m})}^{\alpha}d\theta_{m}$$

$$=\frac{a_{1}\beta^{\alpha}}{\Gamma(\alpha)}*\frac{\Gamma(\alpha+1)}{\beta^{\alpha+1}}\int_{0}^{\infty} \frac{1}{1+c_{1}\theta_{m}}*\frac{\beta^{\alpha+1}}{\Gamma(\alpha+1)}{*e}^{-\beta(\theta_{m})}{{(\theta}_{m})}^{(\alpha+1)-1}d\theta_{m}$$

Since $\Gamma\left( \alpha+1 \right)=\alpha\Gamma\left( \alpha\right)$, gamma distribution$: g\left( \theta_{m} \right)=\frac{\beta^{\alpha}}{\Gamma(\alpha)}e^{-\beta{(\theta}_{m})}{{(\theta}_{m})}^{\alpha-1}$, and $E\left( f(x) \right)=\int_{0}^{\infty} f(x)g\left( \theta_{m} \right)d\theta_{m}$

$$E\left( s_{m} \right)\approx a_{1}\frac{\alpha}{\beta}E(\frac{1}{1+c_{1}\theta_{m}},\alpha+1,\beta)$$

$$=a_{1}\frac{\alpha}{\beta}*\frac{1}{1+c_{1}E(\theta_{m},\alpha+1,\beta)}$$

since the property of gamma distribution: E($\theta_{m})$=$\frac{\alpha}{\beta}$

$$E\left( s_{m} \right)=a_{1}*\frac{\alpha}{\beta}*\frac{1}{1+c_{1}*\frac{\alpha+1}{\beta}}$$

$$=a_{1}*\frac{\alpha}{\beta}*\frac{1}{1+c_{1}*\frac{(\alpha+1)}{\beta}*\frac{\alpha}{\alpha}}$$

$$E\left( s_{m} \right)\approx a_{1}*E\left( \theta_{m} \right)*\frac{1}{1+c_{1}\frac{\left( \alpha+1 \right)}{\alpha}E\left( \theta_{m} \right)} (5)$$

which is consistent with the equation (21) of Tajima 1996 paper used for DNA mutation [[3](#_ENREF_3)].

We assume *π_m_* is the average number of pairwise methylation state differences per cytosine site (note, *π_m_* is different from the average number of pairwise methylation differences, which needs to be divided by the total number of cytosines in a locus to get *π_m_*$)$.

Substituting n=2 into equation (5), we have

$$E\left( \pi_{m} \right)\approx\frac{E\left( \theta_{m} \right)}{1+\frac{2\left( \alpha+1 \right)E\left( \theta_{m} \right)}{\alpha}} (6)$$

From equation (5) and (6), we can get:

$$\hat{\theta_{\pi_{m}}}=\frac{\pi_{m}}{1-2(\alpha+1)\pi_{m}/\alpha} (7)$$

$$\hat{\theta_{s_{m}^{*}}}=\frac{s_{m}^{*}}{a_{1}-c_{1}(\alpha+1)s_{m}^{*}/\alpha} (8)$$

Using $\frac{1}{1-x}\approx exp(x)$ as approximation, the above two equations can become the bellowing:

$$\hat{\theta_{\pi_{m}}}=\pi_{m} exp\left[ \frac{2(\alpha+1)\pi_{m}}{\alpha} \right] (7a)$$

$$\hat{\theta_{s_{m}^{*}}}=\frac{s_{m}^{*}}{a_{1}} exp\left[ \frac{c_{1}(\alpha+1)s_{m}^{*}}{a_{1}\alpha} \right] (8a)$$

Finally, according to Tajima’s D test[[4](#_ENREF_4)],

$$D=\frac{\pi-\frac{S}{a_{1}}}{\sqrt{e_{1}S+e_{2}S(S-1)}}$$

$\pi$ is the average number of nucleotide differences over the whole sequence, and $S$ is the number of segregating sites over the whole sequence. Substituting $\hat{\theta_{\pi_{m}}}*L=\pi$ and $\hat{\theta_{s_{m}}}*L= \frac{s_{m}}{a_{1}}$ (*L* is the length of cytosines in the sequence) into above Tajima’s D test, we developed the neutrality test statistic for methylation mutations as:

$$D^{m}=\frac{\hat{\theta_{\pi_{m}}}-\hat{\theta_{s_{m}^{*}}}}{\sqrt{a_{1}e_{1}\hat{\theta_{s_{m}^{*}}}/L+e_{2}a_{1}\hat{\theta_{s_{m}^{*}}}(a_{1}\hat{\theta_{s_{m}^{*}}}-1/L)}} (9)$$

$$b_{1}=\frac{n+1}{3\left( n-1 \right)}$$

$$b_{2}=\frac{2(n^{2}+n+3)}{9n(n-1)}$$

$$c_{3}=b_{1}-\frac{1}{a_{1}}$$

$$c_{4}=b_{2}-\frac{n+2}{a_{1}n}+\frac{a_{2}}{a_{1}^{2}}$$

$$e_{1}=\frac{c_{3}}{a_{1}}$$

$$e_{2}=\frac{c_{4}}{{a_{1}^{2}+a}_{2}}$$

In Summary, following the framework of the original and modified Tajima’s D test [[3-5](#_ENREF_3)], we derived the equations to compute $\hat{\theta_{\pi_{m}}}$ and $\hat{\theta_{s_{m}}}$ which are suitable for the two states of DNA methylation, and developed the D^m^ test which is a neutrality test for epimutation.

**The commands and parameters for running “ms” and “ms-sel” in the simulations:**

1. neutral scenario without recombination and demographic effects.

msdir/ms 60 10000 -T | grep -v // > treefile

1. demographic effects:

*100 times* *population expansion:*

`msdir/ms 60 10000 -T -eN time 0.01 | grep -v // > treefile`

Here, “time” takes the values: 0, 0.001, 0.01, 0.05, 0.1, 0.2, 0.3, 0.4, 0.5, 0.6, 0.7 and 0.8

*100 times* *population shrinkage:*

`msdir/ms 60 10000 -T -eN time 100 | grep -v // > treefile`

Here, “time” takes the values: 0, 0.001, 0.01, 0.05, 0.1, 0.2, 0.3, 0.4, 0.5, 0.6, 0.7, 0.8, 1, 1.5, 2, 3, 5 and 10

*Population division with 4Nm=0.1*

`msdir/ms 60 10000 -T -I 2 S1 S2 0.1 | grep -v // > treefile`;

Here, the sampling scheme (“S1” and “S2”) takes the values: (0,60), (2,58), (5,55), (10, 50), (15, 45), (20, 40), (25,35), (30,30).

1. selection scenarios:

`mssel 60 10000 anc_pop dec_pop trajectory_file 500 -T -r rec 1000 > sel_tree`

`mssel 60 10000 anc_pop dec_pop trajectory_file 2000 -T -r rec 4000 > sel_tree`

Here, rec=0.8*10^-3^*(1000-1)= 0.7992, anc_pop and dec_pop are the numbers of the two epialleles/alleles sampled, which should equal to the equilibrium frequency of the two epialleles/alleles. Trajectory_file is the frequency trajectory of the epiallele whose frequency increases in the population as time goes.

**Reference**

1. Kimura M (1968) Genetic Variability Maintained in a Finite Population Due to Mutational Production of Neutral and Nearly Neutral Isoalleles. Genetical Research 11: 247-&.

2. Ewens WJ (1972) Sampling Theory of Selectively Neutral Alleles. Theoretical Population Biology 3: 87-&.

3. Tajima F (1996) The amount of DNA polymorphism maintained in a finite population when the neutral mutation rate varies among sites. Genetics 143: 1457-1465.

4. Tajima F (1989) Statistical-Method for Testing the Neutral Mutation Hypothesis by DNA Polymorphism. Genetics 123: 585-595.

5. Misawa K, Tajima F (1997) Estimation of the amount of DNA polymorphism when the neutral mutation rate varies among sites. Genetics 147: 1959-1964.

Supplementary materials S2

The simulation of epigenetic data:

**The inheritance model**:

An epigenetic mark could be lost or gained in one generation.

At a specific site, let 1 denote the presence of a mark, e.g. methylated, and let 0 denote the absence of a mark, e.g. un-methylated.

Assume the loss rate is $\gamma$, namely, the probability that this site with an epigenetic mark in current generation gives rise to this site lacking the mark in the next generation.

The gain rate is $\delta$, namely, the probability that this site without an epigenetic mark in current generation gives rise to this site bearing the mark in the next generation.

***Our model:***

Hence, the inheritance of epigenetic mark can be modeled using a two-state Markov chain. The transition rate matrix, describing the instantaneous rate of change between 0 and 1, is

0 1

$Q=\left\{ q_{ij} \right\}= \begin{matrix} 0 \\ 1 \end{matrix} \left[ \begin{matrix} -\delta& \delta\\ \gamma& -\gamma\end{matrix} \right]$

The diagonals $q_{ii}$ are specified by the requirement that each row of Q sums to zero [[1](#_ENREF_1)].

The Q matrix determines the dynamics of the Markov chain. It can determine the transition-probability matrix over any time t > 0:

$P\left( t \right)=\{p_{ij}\left( t \right)\}$, where $p_{ij}\left( t \right)$is the probability that when t=0, the site is i status, and after t, the site is j status.

$$\frac{dP(t)}{dt}=P\left( t \right)Q$$

Thus, $P\left( t \right)= e^{Qt}$, with the boundary condition $P\left( 0 \right)=I$, the identity matrix (when t=0, no transition occurs).

If the Markov chain has the initial distribution $\pi^{(0)}=(\pi_{0}^{\left( 0 \right)}, \pi_{1}^{\left( 0 \right)})$, then time t later, the distribution $\pi^{(t)}=\left( \pi_{0}^{\left( t \right)}, \pi_{1}^{\left( t \right)} \right)$will be given by

$\pi^{(t)}=\pi^{\left( 0 \right)}P(t)$

Therefore, the model of epigenetic inheritance is analogous to the model of DNA substitution in the finite sites.

For example, the simplest model is when $\gamma=\delta=\mu$per generation.

And $\pi^{(0)}=(0.5, 0.5)$ (the frequency of the site with epigenetic mark = the frequency of the site without epigenetic mark).

$Q=\left\{ q_{ij} \right\}=\left[ \begin{matrix} -\mu& \mu\\ \mu& -\mu\end{matrix} \right]$ and $P_{ij}\left( t \right)=\left\{ \begin{aligned} \frac{1}{2}+\frac{1}{2}e^{-2\mu t} if i=j \\ \frac{1}{2}-\frac{1}{2}e^{-2\mu t} if i\neq j \end{aligned} \right.$

***Slatkin’s model*** (Slatkin 2009)***:***

Given the state of an epigenetic site as a two-state Markov chain, the transition matrix in a single generation is:

$$\Pr\left( 1\to1 \right)= 1- \gamma,\Pr\left( 1\to0 \right)=\gamma,\Pr\left( 0\to1 \right)=\delta,\Pr\left( 0\to0 \right)=1-\delta$$

Thus, the equilibrium frequency of a mark is $\hat{\pi}=\delta/{(\delta+\gamma)}$ and the transition probabilities after *m* generations are:

$\Pr\left( 1,t=m | 1,t=0 \right)={(1-\gamma-\delta)}^{m}+\hat{\pi}[1-\left( 1-\gamma-\delta\right)^{m}]$

$$\Pr\left( 0,t=m | 1,t=0 \right)=\left( 1-\hat{\pi} \right)[1-\left( 1-\gamma-\delta\right)^{m}]$$

$$\Pr\left( 1,t=m | 0,t=0 \right)=\hat{\pi}[1- \left( 1-\gamma-\delta\right)^{m}]$$

$$\Pr\left( 0,t=m | 0,t=0 \right)=\left( 1-\gamma-\delta\right)^{m}+\left( 1-\hat{\pi} \right)[1-\left( 1-\gamma-\delta\right)^{m}]$$

If $\gamma=\delta=\mu$

$$\Pr\left( j,t=m | i,t=0 \right)=\left\{ \begin{aligned} \frac{1}{2}+\frac{1}{2}\left( 1-2\mu\right)^{m} if i=j \\ \frac{1}{2}-\frac{1}{2}\left( 1-2\mu\right)^{m}if i\neq j \end{aligned} \right.$$

When $\mu$ is small, using $1-2\mu\approx e^{-2\mu}$, the above equation can approximate

$\Pr\left( j,t=m | i,t=0 \right)=\left\{ \begin{aligned} \frac{1}{2}+\frac{1}{2}e^{-2\mu m} if i=j \\ \frac{1}{2}-\frac{1}{2}e^{-2\mu m}if i\neq j \end{aligned} \right.$ , which is the same as our model.

Thus, the difference between our model and Slatkin’s model is when $\gamma\neq\delta$. We also simulated the sequences of epigenetic states for this case.

We simulated the neutral scenarios (no recombination, no selection, and no demographic effects), assuming *N_e_* = 1000 and the methylation gain ($\delta$) and loss ($\gamma$) rate taking the values listed in the Table S1 and following a gamma distribution with shape parameter $\alpha=0.2$ among the sites. We did not assume the scenarios when δ/(δ+γ) < 0.5, because there are only two methylation states and those scenarios are symmetric to the ones with δ/(δ+γ) > 0.5.

We plot the expected $\hat{\theta}=4*N_{e}*(\hat{\pi_{0}}*\delta_{0\to1}+\hat{\pi_{1}}*\gamma_{1\to0})$, and $\hat{\theta}_{s}$ and $\hat{\theta}_{\pi}$ estimated by D^m^, D^mod^ and D tests (Figure S1). We found the values estimated by D^m^ were closer to the expectation than those estimated by D^mod^ and D (Figure S1). We also plot the test statistic values, i.g. D^m^, D^mod^ and D (Figure S2). We found D^m^ was closer to zero than D^mod^ and D (Figure S2). Thus, the epigenetic sequences generated by Slatkin’s model and our previous model reach the same conclusion that D^m^ performed better than D^mod^ and D.

***For the situations that initial methylation and unmethylation frequencies are not equal:***

*Equal methylation gain and loss rate*

In the model with equal methylation gain and loss rate, when we changed the initial frequency of methylation and unmethylation, the transition probability matrix *P* will be the same as our previous one. We simulated the epigenetic sequences for these cases in neutral scenarios (no recombination, no selection, and no demographic effects), assuming $\hat{\theta}=0.1$ following a gamma distribution with $\alpha=0.2$ among the sites, and the initial frequency of methylation or unmethylation state as 0.01, 0.02, 0.05, 0.1, 0.2, 0.3, 0.4, and 0.5.

We plot $\hat{\theta}_{s}$ and $\hat{\theta}_{\pi}$ estimated by D^m^, D^mod^ and D tests, and found the values estimated by D^m^ were closer to the expectation (0.1) than those estimated by D^mod^ and D (Figure S3). We also plot the test statistic values, i.g. D^m^, D^mod^ and D, and found D^m^ was closer to zero than D^mod^ and D (Figure S3).

*Methylation gain and loss rate depending on equilibrium methylation and unmethylation frequency*

In the model where the methylation gain and loss rate depending on the epimutation rate and equilibrium methylation and unmethylation frequency, when we change the initial and equilibrium methylation and unmethylation frequency, the transition probability matrix *P* will change.

$\Pr\left( 1,t=m | 1,t=0 \right)=\hat{\pi_{1}}+\left( 1-\hat{\pi_{1}} \right)e^{-1/(2*\hat{\pi_{0}}*\hat{\pi_{1}})\mu t}$

$$\Pr\left( 0,t=m | 1,t=0 \right)=\hat{\pi_{0}}[1-e^{-1/(2*\hat{\pi_{0}}*\hat{\pi_{1}})\mu t}]$$

$$\Pr\left( 1,t=m | 0,t=0 \right)=\hat{\pi_{1}}[1- e^{-1/(2*\hat{\pi_{0}}*\hat{\pi_{1}})\mu t}]$$

$$\Pr\left( 0,t=m | 0,t=0 \right)=\hat{\pi_{0}}+\left( 1-\hat{\pi_{0}} \right)e^{-1/(2*\hat{\pi_{0}}*\hat{\pi_{1}})\mu t}$$

We simulated the epigenetic sequences for these cases in neutral scenarios (no recombination, no selection, and no demographic effects), assuming $\hat{\theta}=0.1$ following a gamma distribution with $\alpha=0.2$ among the sites, and the initial frequency of methylation or unmethylation state as 0.1, 0.2, 0.3, 0.4, and 0.5.

We plot $\hat{\theta}_{s}$ and $\hat{\theta}_{\pi}$ estimated by D^m^, D^mod^ and D tests, and found the values estimated by D^m^ were closer to the expectation (0.1) than those estimated by D^mod^ and D (Figure S4). We also plot the test statistic values, i.g. D^m^, D^mod^ and D, and found D^m^ was closer to zero than D^mod^ and D (Figure S4).

**For neutral scenario**

Assume we sample one allele from 60 samples. We first generated the genealogy tree of the 60 samples with the coalescent algorithm using *ms* package. The branch length of the tree is the evolution time, T, between the ancestor and its child nodes, where T was measured in the unit of *4N* generation ($T=t/4N$). Then we modified the “seq-gen” package [[2](#_ENREF_2)] to simulate the sequence of epigenetic states (marked, unmarked), evolving along the above genealogy tree. First, the oldest ancestral sequences were generated with the given $\pi^{(0)}$in each site of sequence. Then the ancestral sequence was allowed to evolve (gain, loss, and maintain of epigenetic mark) to produce sequences at its daughter nodes. We set a scale factor of the branch length as $\theta=4N\mu$. Thus, after scaling, the length of a branch will become $T*\theta=\left( \frac{t}{4N} \right)*4N\mu=t\mu$. Substituting $t\mu$ into $P_{ij}\left( t \right)=\{p_{ij}\left( t \right)\}$ as shown above, each epigenetic status can be simulated for every site of the child sequence independently. For example, if a site was marked, 1, in the ancestral sequence, the epigenetic status of this site in the child sequence will be a random draw of 0 or 1 with the probabilities, $p_{10}\left( t \right), p_{11}\left( t \right)$ respectively. If the site-specific rate heterogeneity was selected, for example, the rate following a gamma distribution, a different $P_{ij}\left( t \right)$ will be calculated for each rate corresponding to each site. The procedure is repeated for every branch of the tree sequentially, generating the sequence of a node only after the sequence of the ancestral node has been generated. Sequences at the tips of the tree are the final data set.

**For selection scenario**

We adopted the population-epigenetic models of selection developed by Geoghegan and Spencer [[3](#_ENREF_3)].

In the model 1, it assumes a single autosomal locus A, which has two epialleles residing in two different environments (j=1,2) as A_1_ and A_2_.

The corresponding epiallele frequencies are p_1_ and p_2_, respectively, where $\sum p_{k}=1$.

Let G_i_, i= 1,2,3 be the three possible epigenotypes: A_1_A_1_, A_1_A_2_ and A_2_A_2_, respectively. Let v_ij_ be the fitness of individuals of epigenotype G_i_, residing in environment j.

The frequency of environment 1 is r, and that of environment 2 is 1-r.

Let t_j_ be the epigenetic resetting probability to the epigenetic state corresponding to the environment j in which it currently resides. Namely let t_1_ be the probability that A_2_🡪A_1_ in environment 1, and t_2_ be the probability that A_1_🡪A_2_ in environment 2. Thus, if the epiallele bypass epigenetic resetting, affecting the phenotype of the F_1_ generation, despite influences from its current environment j is 1-t_j_.

The un-normalized post-selection frequencies of epialleles A_1_ and A_2_ are:

$p_{1}^{s}=rp_{1}(p_{1}v_{11}+p_{2}v_{21})+t_{1}rp_{2}\left( p_{1}v_{21}+p_{2}v_{31} \right)+(1-t_{2})(1-r)p_{1}(p_{1}v_{12}+p_{2}v_{22})$

and

$$p_{2}^{s}=\left( 1-r \right)p_{2}\left( p_{1}v_{22}+p_{2}v_{32} \right)+t_{2}\left( 1-r \right)p_{1}\left( p_{1}v_{12}+p_{2}v_{22} \right)+$$

$$(1-t_{1})rp_{2}(p_{1}v_{21}+p_{2}v_{31})$$

the recursion equations in the next generation are:

$$p_{1}^{'}={p_{1}^{s}}/\bar{w}$$

and

$$p_{2}^{'}={p_{2}^{s}}/\bar{w}$$

$\bar{w}$ is the mean fitness of the population: $\bar{w}=p_{1}^{s}+p_{2}^{s}$

In the model 2, it assumes two alleles at a single locus, which bears two epialleles inherited from parents residing in two different environments (j = 1, 2); therefore let A_j_ and a_j_ be epialleles A and a, respectively, inherited from environment j. Suppose the epiallele frequencies of A_1_, A_2_, a_1_ and a_2_ are respectively, $p_{1}, p_{2}, p_{3}$ and $p_{4}$, where $\sum p_{k}=1$. Let $G_{i}$, I = 1,2, … , 10 correspond to the ten possible epigenotypes; A_1_A_1_, A_1_A_2_, A_1_a_1_, A_1_a_2_, A_2_A_2_, A_2_a_1_, A_2_a_2_, a_1_a_1_, a_1_a_2_ and a_2_a_2_, respectively. Let v_ij_ be the fitness of individuals of epigenotypes G_i_ residing in environment j. Similar to the model 1, let the frequency of environment 1 be given by r and thus that of environment 2 is 1-r. For simplicity, it assumes that $t_{1}=t_{2}=t$. The un-normalized post-selection frequencies of epialleles A_1_, A_2_, a_1_ and a_2_ are:

$$p_{1}^{s}=rp_{1}\left( p_{1}v_{11}+p_{2}v_{21}+p_{3}v_{31}+p_{4}v_{41} \right)+ trp_{2}\left( p_{1}v_{21}+p_{2}v_{51}+p_{3}v_{61}+p_{4}v_{71} \right)+(1-t)(1-r)p_{1}(p_{1}v_{12}+p_{2}v_{22}+p_{3}v_{32}+p_{4}v_{42})$$

$$p_{2}^{s}=\left( 1-r \right)p_{2}\left( p_{1}v_{22}+p_{2}v_{52}+p_{3}v_{62}+p_{4}v_{72} \right)+t\left( 1-r \right)p_{1}\left( p_{1}v_{12}+p_{2}v_{22}+p_{3}v_{32}+p_{4}v_{42} \right)+\left( 1-t \right)rp_{2}(p_{1}v_{21}+p_{2}v_{51}+p_{3}v_{61}+p_{4}v_{71})$$

$$p_{3}^{s}=rp_{3}\left( p_{1}v_{31}+p_{2}v_{61}+p_{3}v_{81}+p_{4}v_{91} \right)+trp_{4}\left( p_{1}v_{41}+p_{2}v_{71}+p_{3}v_{91}+p_{4}v_{101} \right)+(1-t)(1-r)p_{3}(p_{1}v_{32}+p_{2}v_{62}+p_{3}v_{82}+p_{4}v_{92})$$

$$p_{4}^{s}=\left( 1-r \right)p_{4}\left( p_{1}v_{42}+p_{2}v_{72}+p_{3}v_{92}+p_{4}v_{102} \right)+t\left( 1-r \right)p_{3}\left( p_{1}v_{32}+p_{2}v_{62}+p_{3}v_{82}+p_{4}v_{92} \right)+\left( 1-t \right)rp_{4}(p_{1}v_{41}+p_{2}v_{71}+p_{3}v_{91}+p_{4}v_{101})$$

respectively, hence the recursion equations in the next generation are:

$p_{1}^{'}=p_{1}^{s}/\bar{w}$,

$p_{2}^{'}=p_{2}^{s}/\bar{w}$,

$p_{3}^{'}=p_{3}^{s}/\bar{w}$,

and

$p_{4}^{'}=p_{4}^{s}/\bar{w}$ ,

in which $\bar{w}$ , the mean fitness of the population, is given by

$$\bar{w}=p_{1}^{s}+p_{2}^{s}+p_{3}^{s}+p_{4}^{s}$$

The equilibrium solutions of the above equation are algebraically complicated, and thus, Geoghegan and Spencer explored the parameter space numerically. Based on their results, we picked up several parameter settings, which could lead to the equilibrium status (as shown in Figure 6 of their paper). For the model 1, we picked the fitness set of $v_{11}=0.54, v_{21}=0.51, v_{31}=0.68, v_{12}=0.30, v_{22}=0.45,v_{32}=0.89.$ $p_{1}^{0}=0, p_{2}^{0}=1.$a) with r= 0.5: t = 0.1, t= 0.5 and t=0.9 ; and b) with t=0.5, r=0.1, r=0.5, and r=0.9. Then, we generated the frequency trajectories of $A_{1}$ and $A_{2}$. For the model 2, we picked the fitness set of $v_{11}=0.93, v_{21}=0.26, v_{31}=0.47, v_{41}=0.53, v_{51}=0.98, v_{61}=0.50, v_{71}=0.45, v_{81}=0.78,v_{91}=0.57,v_{101}=0.10,v_{12}=0.88, v_{22}=0.10, v_{32}=0.28, v_{42}=0.16,v_{52}=0.58,v_{62}=0.45,v_{72}=0.23,v_{82}=0.45, v_{92}=0.05, v_{102}=0.90.$ $p_{1}^{0}=0.2, p_{2}^{0}=0, p_{3}^{0}=0.8,$ and $p_{4}^{0}=0$, with t= 0.2, r=0.1 and r=0.33.

Then, we generated the frequency trajectories of $A_{1}, A_{2}, a_{1}$ and $a_{2}$. We merged the frequency trajectories of $A_{1}, A_{2}$, and got the trajectory of allele A. We merged the frequency trajectories of $a_{1}, a_{2}$, and got the trajectory of allele a. We merged those of $A_{1}, a_{1}$, and got that of epiallele 1. We merged those of $A_{2}, a_{2}$, and obtained that of epiallele 2. If the allele/epiallele has frequency larger than 0 at the beginning, we used the trajfixconst and lastp0 scripts in *ms_sel* package to generate the frequency trajectory of allele before that, and used the scripts for the model 1 to generate the frequency trajectory of epiallele before that. Based on the frequency trajectory, we generated the geneology trees of 60 samples of the two epialleles/alleles with proportion of the two epialleles/alleles equal to their equilibrium frequencies, using *ms_sel* package based on coalescent algorithm. Because the genealogy tree of selection favored site and its linked sites that described the states in the coalescence processes and the interval time between different states, could be coalescently determined based on the distribution of ancestral frequencies of selected alleles, namely frequency trajectory here [[4](#_ENREF_4)]. And the effect of recombination could also be included into the simulation of genealogy tree[[5](#_ENREF_5)]. Then, we used the modified “seq-gen” to simulate epigenetic sequences inherited along the genealogy trees of epialleles. And, we used the original “seq-gen” to simulate the genetic sequences inherited along the genealogy trees of alleles. Thus, we generated the epigenetic/genetic sequences under the population epigenetic selection models.

1. Yang Z (2006) Computational molecular evolution. Oxford: Oxford University Press. xvi, 357 p. p.

2. Rambaut A, Grassly NC (1997) Seq-Gen: an application for the Monte Carlo simulation of DNA sequence evolution along phylogenetic trees. Comput Appl Biosci 13: 235-238.

3. Geoghegan JL, Spencer HG (2012) Population-epigenetic models of selection. Theoretical Population Biology 81: 232-242.

4. Kaplan NL, Darden T, Hudson RR (1988) The Coalescent Process in Models with Selection. Genetics 120: 819-829.

5. Hudson RR, Kaplan NL (1988) The Coalescent Process in Models with Selection and Recombination. Genetics 120: 831-840.

Figure S1. Comparison of the $\theta_{m}$ estimators from the D^m^, D^mod^ and D tests based on the simulated SMP data with Slatkin’s Model to the expected values.

Figure S2. Comparison of the test statistics of the D^m^, D^mod^ and D tests based on the simulated SMP data with Slatkin’s Model.

Figure S3. Comparison of the $\theta_{m}$ estimators and test statistics from the D^m^, D^mod^ and D tests under the model with unequal initial methylation and unmethylation frequency but equal methylation gain and loss rate.

Figure S4. Comparison of the $\theta_{m}$ estimators and test statistics from the D^m^, D^mod^ and D tests under the model with unequal initial methylation and unmethylation frequency and the methylation gain and loss rate depending on equilibrium methylation and unmethylation frequency.

Figure S5. The frequency trajectory of the A_1_ epiallele in the three types of scenarios under the population epigenetic selection Model 1 developed by Geoghegan and Spencer (2012). The Fitness set is: $v_{11}=0.54, v_{21}=0.51, v_{31}=0.68, v_{12}=0.30, v_{22}=0.45,v_{32}=0.89$. For the type 1 scenarios: r=0.1, t=0.5, initial p_1_=1, p_2_=0; r=0.5, t=0.1, initial p_1_=1, p_2_=0; and r=0.9, t=0.5, initial p_1_=0, p_2_=1. The type 2 scenarios: r=0.1, t=0.5, initial p_1_=0, p_2_=1; r=0.5, t=0.1, initial p_1_=0, p_2_=1; and r=0.9, t=0.5, initial p_1_=1, p_2_=0. The type 3 scenarios: r=0.5, t=0.5, initial p_1_=1, p_2_=0; r=0.5, t=0.5, initial p_1_=0, p_2_=1; r=0.5, t=0.9, initial p_1_=0, p_2_=1; and r=0.5, t=0.9, initial p_1_=1, p_2_=0.

Figure S6. The frequency trajectories of the allele A & a, and epiallele 1 & 2 in the two types of scenarios under the population epigenetic selection model 2 developed by Geoghegan and Spencer (Model 2). The fitness set of $v_{11}=0.93, v_{21}=0.26, v_{31}=0.47, v_{41}=0.53, v_{51}=0.98, v_{61}=0.50, v_{71}=0.45, v_{81}=0.78,v_{91}=0.57,v_{101}=0.10,v_{12}=0.88, v_{22}=0.10, v_{32}=0.28, v_{42}=0.16,v_{52}=0.58,v_{62}=0.45,v_{72}=0.23,v_{82}=0.45, v_{92}=0.05, v_{102}=0.90.$The initial A_1_, A_2_, a_1_ and a_2_ frequency: $p_{1}^{0}=0.2, p_{2}^{0}=0, p_{3}^{0}=0.8,$ and $p_{4}^{0}=0$, with t= 0.2. The type I scenario: r=0.1 and the type II scenario: r=0.33.

Figure S7. The D^m^, D^mod^ and D test powers in detecting selection based on the SMP data simulated under the population epigenetic selection model 1, with the mixture of neutral loci and selective loci as the null distribution. The ‘x’ axis is the time period after epialleles arrive at the stable equilibrium, measured in 4*N* generations. The ‘y’ axis is the test power. ^a^: The test power was computed as the proportion of test statistic values falling into the lower 5% tail of the null distribution. ^b^: The test power was computed as the proportion of test statistic values falling into the higher 5% tail of the null distribution. See the main text for the description of the three types of scenarios.

Table S1. The methylation gain ($\delta$) and loss ($\gamma$) rate applied in the simulations of Slatkin’s model.

| γ | 2.50E-07 | 5.00E-07 | 1.25E-06 | 2.50E-05 | 5.00E-06 | 7.50E-06 | 1.00E-05 | 1.25E-05 |
| --- | --- | --- | --- | --- | --- | --- | --- | --- |
| δ | 2.475E-05 | 2.45E-05 | 2.375E-05 | 2.25E-05 | 2.00E-05 | 1.75E-05 | 1.50E-05 | 1.25E-05 |
| δ/(δ+γ | 0.99 | 0.98 | 0.95 | 0.90 | 0.80 | 0.70 | 0.60 | 0.50 |

Table S2. The means of $\hat{\theta_{s}}$ and $\hat{\theta_{\pi}}$ from the three tests based on 10000 simulation data under the neutral scenario

|  | $\hat{\theta_{s}}$ | | | $\hat{\theta_{\pi}}$ | | | | | |  |
| --- | --- | --- | --- | --- | --- | --- | --- | --- | --- | --- |
| $\alpha$ | D^m^ | D^mod^ | D | D^m^ | | D^mod^ | | D | | |
|  |  |  | $\theta$=0.005 | | |  | |  | | |
| 10 | 0.004988 | 0.004938 | 0.004903 | 0. 005003 | | 0.004979 | | 0.004933 | | |
| 1 | 0.005010 | 0.004920 | 0.004858 | 0.005032 | | 0.004989 | | 0.004906 | | |
| 0.5 | 0.005034 | 0.004901 | 0.004809 | 0.005076 | | 0.005012 | | 0.004887 | | |
| 0.2 | 0.005064 | 0.004809 | 0.004636 | 0.005103 | | 0.004979 | | 0.004743 | | |
| 0.1 | 0.005073 | 0.004640 | 0.004358 | 0.005172 | | 0.004954 | | 0.004551 | | |
|  |  |  | $\theta$=0.01 | | |  | |  | | |
| 10 | 0.009986 | 0.009795 | 0.009661 | 0.010029 | | 0.009939 | | 0.009762 | | |
| 1 | 0.010054 | 0.009712 | 0.009478 | 0.010124 | | 0.009961 | | 0.009647 | | |
| 0.5 | 0.010056 | 0.009563 | 0.009229 | 0.010172 | | 0.009934 | | 0.009478 | | |
| 0.2 | 0.010118 | 0.009212 | 0.008622 | 0.010269 | | 0.009820 | | 0.008991 | | |
| 0.1 | 0.010190 | 0.008723 | 0.007820 | 0.010519 | | 0.009751 | | 0.008404 | | |
|  |  |  | $\theta$=0.02 | | |  | |  | | |
| 10 | 0.020013 | 0.019283 | 0.018782 | 0.020174 | | 0.019828 | | 0.019157 | | |
| 1 | 0.020103 | 0.018846 | 0.018006 | 0.020386 | | 0.019775 | | 0.018618 | | |
| 0.5 | 0.020109 | 0.018339 | 0.017185 | 0.020514 | | 0.019634 | | 0.018006 | | |
| 0.2 | 0.020236 | 0.017186 | 0.015320 | 0.020927 | | 0.019331 | | 0.016541 | | |
| 0.1 | 0.020340 | 0.015747 | 0.013155 | 0.021667 | | 0.019051 | | 0.014819 | | |
|  |  |  | $\theta$=0.05 | | |  | |  | | |
| 10 | 0.049698 | 0.045700 | 0.043072 | 0.050761 | | 0.048786 | | 0.045099 | | |
| 1 | 0.049702 | 0.043292 | 0.039275 | 0.051513 | | 0.048192 | | 0.042261 | | |
| 0.5 | 0.049664 | 0.041114 | 0.035988 | 0.052162 | | 0.047544 | | 0.039625 | | |
| 0.2 | 0.049736 | 0.036568 | 0.029453 | 0.054181 | | 0.046326 | | 0.034104 | | |
| 0.1 | 0.049716 | 0.031863 | 0.023310 | 0.057406 | | 0.045293 | | 0.028548 | | |
|  |  |  | $\theta$=0.1 | | |  | |  | | |
| 10 | 0.096492 | 0.083515 | 0.075419 | 0.101262 | | 0.094411 | | 0.082219 | | |
| 1 | 0.095917 | 0.076522 | 0.065252 | 0.103347 | | 0.092328 | | 0.073972 | | |
| 0.5 | 0.095651 | 0.071010 | 0.057562 | 0.105719 | | 0.090787 | | 0.067346 | | |
| 0.2 | 0.095039 | 0.060421 | 0.043923 | 0.112096 | | 0.087904 | | 0.054656 | | |
| 0.1 | 0.094342 | 0.050749 | 0.032816 | 0.121610 | | 0.085722 | | 0.043350 | | |
|  |  |  | $\theta$=0.2 | | |  | |  | | |
| 10 | 0.175808 | 0.140370 | 0.119726 | | 0.196763 | | 0.175518 | 0.119726 |  |  |
| 1 | 0.174849 | 0.124921 | 0.098523 | | 0.204222 | | 0.171053 | 0.098523 |  |  |
| 0.5 | 0.174289 | 0.113506 | 0.083867 | | 0.211676 | | 0.167797 | 0.083867 |  |  |
| 0.2 | 0.173122 | 0.093370 | 0.060420 | | 0.231833 | | 0.162902 | 0.060420 |  |  |
| 0.1 | 0.171215 | 0.076237 | 0.043132 | | 0.258341 | | 0.159035 | 0.043132 |  |  |

Table S3. The means and standard deviations of D^m^, D^mod^, and D under the neutral scenario

|  | D^m^ | |  | D^mod^ | | |  | D | |
| --- | --- | --- | --- | --- | --- | --- | --- | --- | --- |
| $\alpha$ | Mean | SD |  | Mean | | SD |  | Mean | SD |
|  |  |  |  | $\theta$=0.005 | | |  |  |  |
| 10 | -0.077672 | 0.919241 |  | -0.061484 | 0.921627 | |  | -0.066298 | 0.911533 |
| 1 | -0.069449 | 0.928258 |  | -0.040313 | 0.932359 | |  | -0.049204 | 0.914145 |
| 0.5 | -0.063509 | 0.941945 |  | -0.020884 | 0.948186 | |  | -0.0341 | 0.920110 |
| 0.2 | -0.063333 | 0.965397 |  | 0.019714 | 0.976981 | |  | -0.008152 | 0.922101 |
| 0.1 | -0.026517 | 0.993851 |  | 0.116232 | 1.013064 | |  | 0.062436 | 0.915858 |
|  |  |  |  | $\theta$=0.01 | | |  |  |  |
| 10 | -0.082759 | 0.910871 |  | -0.048712 | | 0.916758 |  | -0.058429 | 0.897521 |
| 1 | -0.078323 | 0.921711 |  | -0.01761 | | 0.932269 |  | -0.035763 | 0.897072 |
| 0.5 | -0.060492 | 0.937093 |  | 0.028114 | | 0.951910 |  | 0.000064 | 0.899542 |
| 0.2 | -0.048103 | 0.972240 |  | 0.117088 | | 0.998939 |  | 0.059684 | 0.898832 |
| 0.1 | 0.009187 | 1.019773 |  | 0.284156 | | 1.062721 |  | 0.172022 | 0.890102 |
|  |  |  |  | $\theta$=0.02 | | |  |  |  |
| 10 | -0.077464 | 0.908001 |  | -0.008804 | | 0.920689 |  | -0.028775 | 0.883347 |
| 1 | -0.059961 | 0.932977 |  | 0.059742 | | 0.954742 |  | 0.021845 | 0.887699 |
| 0.5 | 0.038370 | 0.951243 |  | 0.133156 | | 0.981681 |  | 0.0747 | 0.884590 |
| 0.2 | 0.013480 | 1.003285 |  | 0.320091 | | 1.054651 |  | 0.197490 | 0.875690 |
| 0.1 | 0.116174 | 1.069658 |  | 0.603430 | | 1.15001 |  | 0.365137 | 0.856019 |
|  |  |  |  | $\theta$=0.05 | | |  |  |  |
| 10 | -0.036111 | 0.932263 |  | 0.125592 | | 0.962222 |  | 0.072243 | 0.875075 |
| 1 | 0.016720 | 0.968638 |  | 0.286146 | | 1.016453 |  | 0.183747 | 0.868554 |
| 0.5 | 0.065501 | 0.998853 |  | 0.437843 | | 1.063366 |  | 0.280 | 0.856138 |
| 0.2 | 0.203444 | 1.075364 |  | 0.823527 | | 1.178601 |  | 0.493254 | 0.823254 |
| 0.1 | 0.427448 | 1.17579 |  | 1.354367 | | 1.327607 |  | 0.735312 | 0.778419 |
|  |  |  |  | $\theta$=0.1 | | |  |  |  |
| 10 | 0.066603 | 0.969436 |  | 0.355045 | | 1.018919 |  | 0.239792 | 0.863123 |
| 1 | 0.166824 | 1.014191 |  | 0.626150 | | 1.089793 |  | 0.406809 | 0.838464 |
| 0.5 | 0.263689 | 1.054155 |  | 0.879608 | | 1.153333 |  | 0.543 | 0.812102 |
| 0.2 | 0.523898 | 1.162867 |  | 1.498312 | | 1.315908 |  | 0.819393 | 0.759144 |
| 0.1 | 0.901535 | 1.303849 |  | 2.307729 | | 1.526221 |  | 1.094570 | 0.700920 |
|  |  |  |  | $\theta$=0.2 | | |  |  |  |
| 10 | 0.330203 | 1.019166 |  | 0.801970 | | 1.086596 |  | 0.547906 | 0.832183 |
| 1 | 0.498634 | 1.077018 |  | 1.221139 | | 1.179763 |  | 0.754001 | 0.783655 |
| 0.5 | 0.662074 | 1.129685 |  | 1.606565 | | 1.263168 |  | 0.913 | 0.743028 |
| 0.2 | 1.094997 | 1.286222 |  | 2.539880 | | 1.493317 |  | 1.215158 | 0.675150 |
| 0.1 | 1.682311 | 1.490492 |  | 3.724320 | | 1.793 |  | 1.489546 | 0.614201 |

Table S4. The $\theta_{m}$estimator and test statistics of the three tests in the selection scenarios

|  | Neutral ($\theta_{m}=0.1)$ | | | |
| --- | --- | --- | --- | --- |
|  | $\hat{\theta_{s}}$ | $\hat{\theta_{\pi}}$ | | D |
| D^m^ | 0.0957 | 0.1057 | | 0.2637 |
| D^mod^ | 0.071 | 0.0908 | | 0.8796 |
| D | 0.0576 | 0.0673 | | 0.5435 |
|  | After 15*4N generation | | | |
|  | Type 1 scenario (after 0.1*4N generation) | | | |
|  | $\hat{\theta_{s}}$ | | $\hat{\theta_{\pi}}$ | D |
| D^m^ | 0.0644 | | 0.0360 | -1.59072 |
| D^mod^ | 0.0512 | | 0.0337 | -1.24786 |
| D | 0.0436 | | 0.0296 | -1.16652 |
|  | Type 2 scenario | | | |
|  | $\hat{\theta_{s}}$ | $\hat{\theta_{\pi}}$ | | D |
| D^m^ | 0.1831 | 0.1412 | | -0.8311 |
| D^mod^ | 0.1179 | 0.1181 | | -0.015 |
| D | 0.0864 | 0.0831 | | -0.1481 |
|  | Type 3 scenario | | | |
|  | $\hat{\theta_{s}}$ | $\hat{\theta_{\pi}}$ | | D |
| D^m^ | 0.1803 | 0.3685 | | 3.6496 |
| D^mod^ | 0.1166 | 0.2713 | | 4.6596 |
| D | 0.0857 | 0.1478 | | 2.5664 |
|  | Type I scenario | | | |
|  | $\hat{\theta_{s}}$ | $\hat{\theta_{\pi}}$ | | D |
| D^m^ | 0.1865 | 0.1164 | | -1.3451 |
| D^mod^ | 0.1196 | 0.0993 | | -0.6147 |
| D | 0.0873 | 0.0726 | | -0.6061 |
|  | Type II scenario | | | |
|  | $\hat{\theta_{s}}$ | $\hat{\theta_{\pi}}$ | | D |
| D^m^ | 0.181 | 0.2829 | | 1.9535 |
| D^mod^ | 0.1169 | 0.2169 | | 2.9906 |
| D | 0.0859 | 0.128 | | 1.7315 |

Table S5. The means and standard deviations of D^m^, D^mod^, and D computed with $\alpha$=0.5 based on the SMPs simulated with $\alpha$=0.1, 0.2, 0.5, 1,10

|  | D^m^ | |  | D^mod^ | |  | D | |
| --- | --- | --- | --- | --- | --- | --- | --- | --- |
| $\alpha$ | Mean | SD |  | Mean | SD |  | Mean | SD |
|  |  |  |  | $\theta$=0.02 | |  |  |  |
| 10 | -0.159404 | 0.950604 |  | 0.026759 | 0.988101 |  | -0.028775 | 0.883347 |
| 1 | -0.099879 | 0.955613 |  | 0.079229 | 0.989658 |  | 0.021845 | 0.887699 |
| 0.5 | -0.038370 | 0.951243 |  | 0.133156 | 0.981681 |  | 0.0747 | 0.884590 |
| 0.2 | 0.103767 | 0.939696 |  | 0.257483 | 0.96234 |  | 0.197490 | 0.875690 |
| 0.1 | 0.294641 | 0.914804 |  | 0.427143 | 0.930591 |  | 0.365137 | 0.856019 |
|  |  |  |  | $\theta$=0.05 | |  |  |  |
| 10 | -0.211934 | 1.029905 |  | 0.223446 | 1.126960 |  | 0.072243 | 0.875075 |
| 1 | -0.062373 | 1.017920 |  | 0.340006 | 1.096276 |  | 0.183747 | 0.868554 |
| 0.5 | 0.065501 | 0.998853 |  | 0.437843 | 1.063366 |  | 0.280 | 0.856138 |
| 0.2 | 0.343236 | 0.950971 |  | 0.652658 | 0.991320 |  | 0.493254 | 0.823254 |
| 0.1 | 0.645471 | 0.888620 |  | 0.891916 | 0.910927 |  | 0.735312 | 0.778419 |
|  |  |  |  | $\theta$=0.1 | |  |  |  |
| 10 | -0.202504 | 1.146698 |  | 0.573448 | 1.328270 |  | 0.239792 | 0.863123 |
| 1 | 0.056761 | 1.098998 |  | 0.744378 | 1.230776 |  | 0.406809 | 0.838464 |
| 0.5 | 0.263689 | 1.054155 |  | 0.879608 | 1.153333 |  | 0.543 | 0.812102 |
| 0.2 | 0.663712 | 0.963698 |  | 1.14178 | 1.015174 |  | 0.819393 | 0.759144 |
| 0.1 | 1.034151 | 0.865514 |  | 1.391243 | 0.888705 |  | 1.094570 | 0.700920 |

Table S6. The test powers of D^m^, D^mod^, and D computed with $\alpha$=0.05 and 10 based on the SMPs simulated with $\alpha$=0.5

| r | t | initial | | equilibrium | | scenario type | D^m^ | D^mod^ | D |
| --- | --- | --- | --- | --- | --- | --- | --- | --- | --- |
|  |  | P_1_ | P_2_ | P_1_ | P_2_ |  |  |  |  |
|  |  |  |  | Computed with $\alpha$ = 0.05 | | |  |  |  |
| 0.1 | 0.5 | 1 | 0 | 0.0552 | 0.9448 | 1 | 0.9434 | 0.9999 | 1 |
| 0.5 | 0.1 | 0 | 1 | 0.1093 | 0.8907 | 2 | 0.0447 | 0.0463 | 0.0474 |
| 0.5 | 0.5 | 1 | 0 | 0.413 | 0.587 | 3 | 0.2481 | 0.3348 | 0.3464 |
|  |  |  |  | Computed with $\alpha$ = 10 | | |  |  |  |
| 0.1 | 0.5 | 1 | 0 | 0.0552 | 0.9448 | 1 | 1 | 1 | 1 |
| 0.5 | 0.1 | 0 | 1 | 0.1093 | 0.8907 | 2 | 0.0466 | 0.0472 | 0.0474 |
| 0.5 | 0.5 | 1 | 0 | 0.413 | 0.587 | 3 | 0.3359 | 0.3447 | 0.3464 |

Table S7. The test powers in the three types of scenarios under the population epigenetic selection Model 1 with the mixture of neutral loci and selective loci as the null distribution

| r | t | initial | | equilibrium | | scenario type | D^m^ | D^mod^ | D |
| --- | --- | --- | --- | --- | --- | --- | --- | --- | --- |
|  |  | P_1_ | P_2_ | P_1_ | P_2_ |  |  |  |  |
| 0.5 | 0.1 | 0 | 1 | 0.1093 | 0.8907 | 2 | 0.0073 | 0.0073 | 0.0073 |
| 0.5 | 0.1 | 1 | 0 | 0.1093 | 0.8907 | 1 | 0.9669 | 0.9716 | 0.9669 |
| 0.5 | 0.5 | 0 | 1 | 0.413 | 0.587 | 3 | 0.0278 | 0.0283 | 0.0278 |
| 0.5 | 0.5 | 1 | 0 | 0.413 | 0.587 | 3 | 0.1235 | 0.1288 | 0.1235 |
| 0.5 | 0.9 | 0 | 1 | 0.5103 | 0.4897 | 3 | 0.0627 | 0.0647 | 0.0627 |
| 0.5 | 0.9 | 1 | 0 | 0.5103 | 0.4897 | 3 | 0.0575 | 0.0594 | 0.0575 |
| 0.1 | 0.5 | 0 | 1 | 0.0552 | 0.9448 | 2 | 0.0068 | 0.0063 | 0.0068 |
| 0.1 | 0.5 | 1 | 0 | 0.0552 | 0.9448 | 1 | 0.9999 | 0.9999 | 0.9999 |
| 0.9 | 0.5 | 0 | 1 | 0.9378 | 0.0622 | 1 | 1 | 1 | 1 |
| 0.9 | 0.5 | 1 | 0 | 0.9378 | 0.0622 | 2 | 0.007 | 0.0068 | 0.007 |

Table S8. The annotation of the new genes in *Arabidopsis* and human with significant D^m^ values

| Species | Gene name | D^m^ | P value | Annotation | Function | Involved in | InterPro Domains | Source |
| --- | --- | --- | --- | --- | --- | --- | --- | --- |
| *Arabidopsis* | \| AT1G21530 \| \| --- \| | -2.5601 | 0.0012 | AMP-dependent synthetase and ligase family protein | catalytic activity | metabolic process | AMP-binding | TAIR |
| *Arabidopsis* | AT1G31670 | -2.415 | 0.0231 | Copper amine oxidase family protein | quinone binding, primary amine oxidase activity, copper ion binding | oxidation reduction, amine metabolic process | copper amine oxidase | TAIR |
| *Arabidopsis* | AT1G68280 | -2.5016 | 0.0161 | Thioesterase superfamily protein | NA | NA | Thioesterase superfamily | TAIR |
| *Arabidopsis* | AT1G73607 | -2.6957 | 0.0058 | Encodes a member of a family of small, secreted, cysteine rich protein with sequence similarity to the PCP (pollen coat | NA | NA |  | TAIR |
| *Arabidopsis* | AT2G07715 | -2.3172 | 0.0375 | Nucleic acid-binding, OB-fold-like protein | structural constituent of ribosome | translation | Nucleic acid-binding | TAIR |
| *Arabidopsis* | AT2G43440 | -2.9163 | 0.0021 | F-box and associated interaction domains containing protein | NA | NA | F-box domains | TAIR |
| *Arabidopsis* | AT3G25960 | -2.4697 | 0.0169 | Pyruvate kinase family protein | pyruvate kinase activity, magnesium ion binding, potassium ion binding, catalytic activity | glycolysis | Pyruvate kinase domains | TAIR |
| *Arabidopsis* | AT3G47760 | -2.2924 | 0.0462 | member of ATH subfamily | NA | NA | NA | TAIR |
| *Arabidopsis* | AT4G33320 | -2.2603 | 0.0499 | Plant protein of unknown function | NA | NA | NA | TAIR |
| *Arabidopsis* | AT4G34900 | -2.4413 | 0.021 | xanthine dehydrogenase 2 | 8 functions | oxidation reduction, allantoin biosynthetic process | dehydrogenase domains | TAIR |
| *Arabidopsis* | AT1G33607 | 2.0763 | 0.0445 | Encodes a defensing-like (DEFL) family protein | NA | NA | NA | TAIR |
| *Arabidopsis* | AT4G21460 | 3.6345 | 0.0078 | Ribosomal protein S24/S35 | NA | NA | Ribosomal protein | TAIR |
| Human | C19orf48 | -1.6132 | 0.0688 | Multidrug resistance-related protein | NA | NA | NA | Uniprot |
| Human | FLJ26850 | -1.7224 | 0.0481 | a RNA gene, and is affiliated with the lncRNA class | NA | NA | NA | NCBI,genecards |
